# Single-cell landscape of primary central nervous system diffuse large B-cell lymphoma

**DOI:** 10.1038/s41421-023-00559-7

**Published:** 2023-06-12

**Authors:** Nianping Liu, Chen Jiang, Xinfeng Yao, Minghao Fang, Xiaolong Qiao, Lin Zhu, Zongcheng Yang, Xuyuan Gao, Ying Ji, Chaoshi Niu, Chuandong Cheng, Kun Qu, Jun Lin

**Affiliations:** 1 Department of Neurosurgery, The First Affiliated Hospital of USTC, School of Basic Medical Sciences, Division of Life Sciences and Medicine, University of Science and Technology of China, Hefei, Anhui China; 2 Institute of Artificial Intelligence, Hefei Comprehensive National Science Center, Hefei, Anhui China; 3 Division of Life Sciences and Medicine, University of Science and Technology of China, Hefei, Anhui China; 4 Anhui University of Science and Technology, Huainan, Anhui China; 5 Department of Stomatology, The First Affiliated Hospital of USTC, Division of Life Sciences and Medicine, University of Science and Technology of China, Hefei, Anhui China; 6 CAS Center for Excellence in Molecular Cell Sciences, The CAS Key Laboratory of Innate Immunity and Chronic Disease, University of Science and Technology of China, Hefei, Anhui China

**Keywords:** Cancer genomics, Tumour immunology, CNS cancer, Haematological cancer

## Abstract

Understanding tumor heterogeneity and immune infiltrates within the tumor-immune microenvironment (TIME) is essential for the innovation of immunotherapies. Here, combining single-cell transcriptomics and chromatin accessibility sequencing, we profile the intratumor heterogeneity of malignant cells and immune properties of the TIME in primary central nervous system diffuse large B-cell lymphoma (PCNS DLBCL) patients. We demonstrate diverse malignant programs related to tumor-promoting pathways, cell cycle and B-cell immune response. By integrating data from independent systemic DLBCL and follicular lymphoma cohorts, we reveal a prosurvival program with aberrantly elevated RNA splicing activity that is uniquely associated with PCNS DLBCL. Moreover, a plasmablast-like program that recurs across PCNS/activated B-cell DLBCL predicts a worse prognosis. In addition, clonally expanded CD8 T cells in PCNS DLBCL undergo a transition from a pre-exhaustion-like state to exhaustion, and exhibit higher exhaustion signature scores than systemic DLBCL. Thus, our study sheds light on potential reasons for the poor prognosis of PCNS DLBCL patients, which will facilitate the development of targeted therapy.

## Introduction

Primary central nervous system diffuse large B-cell lymphoma (PCNS DLBCL) is a rare and aggressive non-Hodgkin lymphoma, histologically accounting for the majority (90%) of non-HIV-associated primary central nervous system lymphoma^1^. Recent years have seen significant progress in the treatment of PCNS DLBCL. The MATRix regimen followed by either autologous stem cell transplantation or whole-brain radiotherapy demonstrates its efficacy, achieving approximately up to 80% 2-years progression-free survival^2–4^. However, despite these advances, therapeutic resistance and relapse remain common and contribute to the poor prognosis for PCNS DLBCL patients, with 5-year survival rates of only 30%–40%^5^.

In 2000, Alizadeh et al. categorized DLBCL into two subtypes based on the cell of origin (COO): germinal center B-cell-like DLBCL (GCB-like) and activated B-cell-like (ABC-like) DLBCL. Although PCNS DLBCL is morphologically similar to systemic/extracerebral DLBCL^6^, previous lines of evidence (both gene expression analyses^7,8^ and immunohistochemical analyses^9^) have suggested distinct molecular features and subtypes of PCNS DLBCL compared with systemic DLBCL, as well as extensive intertumoral heterogeneity of immune infiltrates among DLBCL tumors^10^. However, the intratumoral heterogeneity within individual tumors remains unclear in PCNS DLBCL, which has been highlighted by emerging insights into its significant contribution to drug resistance and tumor recurrence^11^.

High-throughput single-cell sequencing technologies offer unprecedented access to assess intratumor heterogeneity and immune infiltrates within the tumor-immune microenvironment (TIME). Recent efforts have been made to resolve the heterogeneity of extracerebral B-cell lymphoma, such as follicular lymphoma (FL)^12,13^ and systemic DLBCL^13–16^, by single-cell RNA sequencing. Notably, checkpoint molecule expression on infiltrating T-cell subsets has been profiled in FL and classic Hodgkin lymphoma^12,17^. However, there are limited studies to resolve the complexities of malignant cells and tumor-infiltrating immune cells in PCNS DLBCL patients at single-cell resolution. For example, Ruan et al. characterized the phenotypic states of ~1000 diffuse large B cells from the cerebrospinal fluid (CSF) of CNS DLBCL patients^15^, but CSF could not completely reflect the composition and transcriptional heterogeneity of the TIME in these patients. Moreover, integrative analysis of PCNS and systemic DLBCL at the single-cell level, which would provide a broader understanding of PCNS DLBCL, is also lacking.

Here, we depicted the landscape of the TIME in patients with PCNS DLBCL by performing single-cell transcriptome and chromatin accessibility assays on patients who underwent surgical resection. We revealed that phenotypically monoclonal or oligoclonal malignant B cells showed aberrant expression programs. For example, a plasmablast-like program was associated with a worse prognosis. In addition, integrative analysis of malignant PCNS DLBCL cells and extracerebral B-cell lymphomas supported the presence of a PCNS DLBCL-specific BCL2-high phenotype with a tumor-promoting feature. Moreover, we observed higher expression levels of exhaustion signatures in tumor-infiltrating CD8 T cells from patients with PCNS DLBCL compared with systemic DLBCL, which may be one of the underlying reasons for the dismal prognosis of PCNS DLBCL patients.

## Results

### Single-cell landscape of PCNS DLBCL

**Fig. 1:**
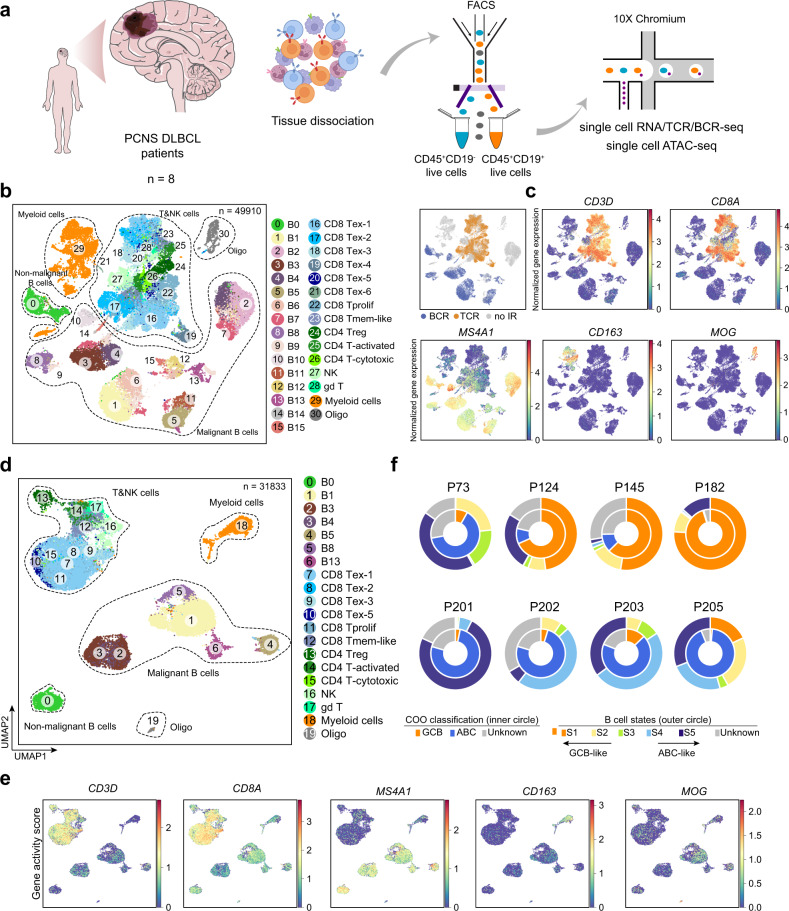
Characterization of the PCNS DLBCL TIME using scRNA-seq paired with V(D)J profiling and scATAC-seq. **a** Schematic of the workflow for tumor section processing and scRNA-seq paired with V(D)J profiling and scATAC-seq. **b** UMAP of all cells colored by cell clusters (left) from scRNA-seq and immune receptor classification (right) based on scV(D)J data. Dashed circles highlight the major cell types. NK natural killer cells, Oligo oligodendrocytes, gd T gamma-delta T cells, Treg regulatory T cells, CD8 Tex exhausted CD8 T cells, CD8 Tprolif proliferative CD8 T cells, CD8 Tmem-like memory-like CD8 T cells. **c** UMAP of all cells colored by normalized expression levels of selected marker genes in the scRNA-seq data. Color bars indicate the log-normalized expression values of each gene in single cells. **d** UMAP of all cells colored by cell clusters from scATAC-seq. Dashed circles highlight the major cell types. **e** UMAP of all cells colored by gene activity scores of selected marker genes in the scATAC-seq data. Color bars indicate the gene activity scores of each gene in single cells, which were calculated by summing the fragments intersecting with the region of gene body and 2 kb upstream of transcription start site. **f** Donut plot showing the B-cell state classification (outer circle) and COO classification (inner circle) of malignant B cells in each patient. Arrows indicate that B-cell states from S1 to S5 represent malignancy from more GCB-like to more ABC-like subtypes. In (**c**) and (**e**), marker genes included *CD3D* for T cells, *CD8A* for CD8 T cells, *MS4A1* for B cells, *CD163* for myeloid cells, and *MOG* for oligodendrocytes.

To characterize the malignant cells and their TIME of PCNS DLBCL, we performed 5′ single-cell RNA sequencing (scRNA-seq) on CD45^+^CD19^+^ and CD45^+^CD19^−^ immune cells isolated from a cohort of 8 immunocompetent patients (two replicates for P73; Supplementary Table S1). Each sample was also examined with paired single-cell T-cell receptor sequencing (scTCR-seq, *n* = 7 patients) and B-cell receptor sequencing (BCR-seq, *n* = 7 patients, two replicates for P73) (Fig. 1a and Supplementary Fig. S1a). In total, we obtained 49,910 high-quality single-cell transcriptomes, with an average of 10,570 unique molecular identifiers, representing 2469 genes (Supplementary Fig. S1b; see “Materials and methods”). The TCR and BCR sequences were assembled by using CellRanger: TCR signals were detected in 16,539 cells, and BCR signals were detected in 14,493 cells. We used Scanpy^18^ to merge and normalize the scRNA-seq profiles and visualized the cells via uniform manifold approximation and projection (UMAP) (Fig. 1b). This analysis indicated four major cell types, including B cells, natural killer (NK) & T cells, myeloid cells, and oligodendrocytes, based on expression levels of canonical marker genes (Fig. 1b, c; see “Materials and methods”). The TCR/BCR profiles were consistent with the identification of major cell types (Fig. 1b). Data for the B cells and NK&T cells were extracted, and we performed a second round of clustering analysis that identified 16 B-cell subtypes and 13 NK&T-cell subtypes (Supplementary Fig. S2; see “Materials and methods”).

We also applied single-cell assay for transposase-accessible chromatin using sequencing (scATAC-seq) to investigate the chromatin accessibility of CD45^+^CD19^+^ and CD45^+^CD19^−^ cells from 5 of the above patients who were subjected to scRNA-seq (Fig. 1a). After filtering out low-quality cells and doublets, we obtained high-quality chromatin accessibility profiles for a total of 31,833 single cells (Supplementary Fig. S1c–e; see “Materials and methods”). We then leveraged the high-resolution annotations of cell populations identified by scRNA-seq to annotate our chromatin accessibility profiles to optimize the representation between these two cross-modality datasets by using Seurat^19^ (Fig. 1d and Supplementary Fig. S3a). The chromatin accessibility analysis of known marker genes distinguished different cell clusters of scATAC-seq data (Fig. 1e and Supplementary Fig. S3a). The Jaccard similarity index also showed that the cluster annotations were consistent with the results of unsupervised clustering (Supplementary Fig. S3b, c), indicating that we obtained a reliable single-cell atlas of chromatin accessibility profiles.

### Characterization of malignant B cells in patients with PCNS DLBCL

To distinguish malignant from nonmalignant B cells, we conducted a single-cell copy number variation (CNV) analysis of the scRNA-seq data using inferCNV^20^. We found no significant CNV for cluster B0, however, other 15 B clusters were heterogeneous in terms of chromosome copy number, displaying canonical CNVs for PCNS lymphomas, such as gain of chromosome 12 and/or loss of chromosomes 6 and 8^21^ (Supplementary Fig. S4a). In addition, all B-cell clusters, except the B0 cluster, significantly expressed a dominant type of immunoglobulin light chain (Supplementary Fig. S4b, c), which was consistent with the allelic exclusion phenotypes of DLBCL tumor cells reported in previous studies^13,22^. These results indicated that the B0 cluster cells are nonmalignant, while the B1–B15 cluster cells are malignant. Moreover, we examined the BCR clonotype of the B cells and found that the malignant B cells in the individual patients presented with monoclonal or rarely oligoclonal phenotypes (Supplementary Fig. S5).

We then applied a gene signature-based classifier^23^ to categorize the COO classification of PCNS DLBCL patients based on our scRNA-seq data. The results indicated that malignant cells from P73, P201, P202, P203, and P205 are dominated by the ABC-like subtype, while malignant cells from P124, P145, and P182 are dominated by the GCB-like subtype (Fig. 1f). Recently, B-cell states were proposed to further clarify the COO hierarchy underlying the GCB/ABC dichotomy^14^; states from S1 to S5 were reported to informatively represent a state transition from GCB-like to ABC-like subtypes. B-cell state classification analysis showed that the B-cell states of the examined patients were consistent with those of the COO classification (Fig. 1f).

### Heterogeneous malignant meta-programs in PCNS DLBCL

**Fig. 2:**
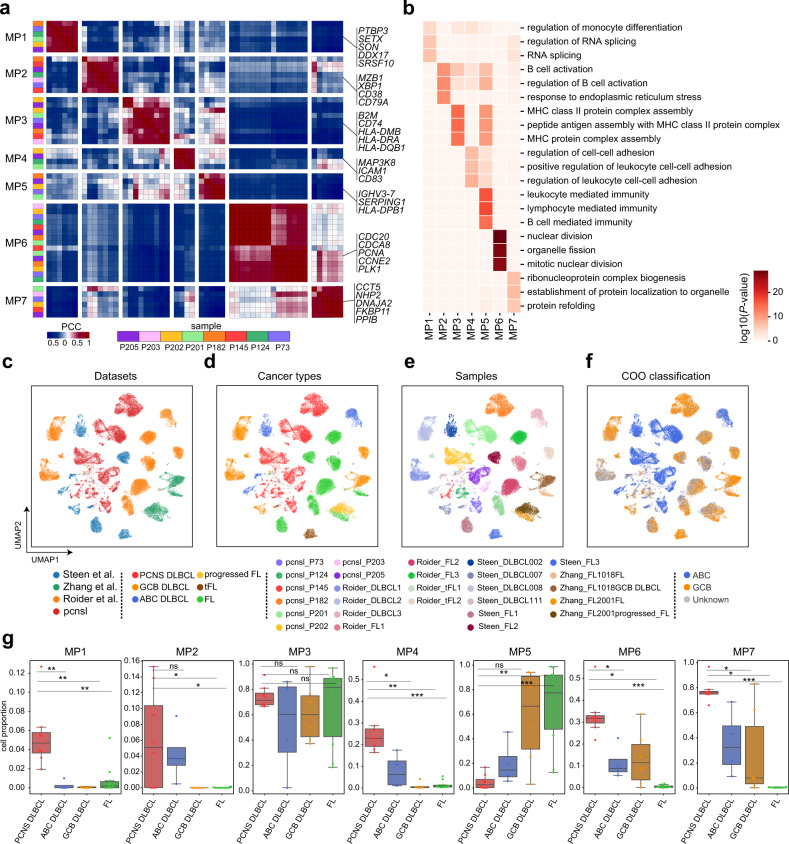
Identification of intratumor malignant meta-programs in PCNS DLBCL patients. **a** Heatmap depicting pair-wise correlations of intratumor programs derived from PCNS DLBCL patients. Both rows and columns represent programs identified by consensus NMF algorithm sample-wise. Color bar on the left indicates which sample each program belongs to. Color of each block represents the Pearson correlation coefficient (PCC) between two programs. Further hierarchical clustering identified seven coherent expression meta-programs across samples (MP1–7). The representative genes of each meta-program are displayed on the right of the heatmap. **b** Heatmap of representative biological pathways significantly enriched in the seven meta-programs. UMAP of malignant cells colored by datasets (**c**), cancer types (**d**), samples (**e**), and COO classification (**f**). FL follicular lymphoma, tFL transformed follicular lymphoma, DLBCL diffuse large B-cell lymphoma. **g** Boxplot showing the proportion of cells enriched for the meta-programs in each patient. Each dot in the boxplot represents the cell proportion of an individual patient, and patients were classified into different cancer types. A two-sided Wilcoxon rank-sum statistic was used to calculate significance (***P < 0.001, **P < 0.01, *P < 0.05). Box boundaries and middle lines correspond to the interquartile range (IQR) and median, respectively. Whiskers extend to the lowest or highest data points that are no more than 1.5 times the IQR from the box boundaries. In (**c**), Steen et al.^14^, Zhang et al.^27^ and Roider et al.^13^ represent three publicly available scRNA-seq datasets; “pcnsl” represents PCNS DLBCL dataset in this study.

To resolve the transcriptional spectrum of intratumor heterogeneity in malignant B cells, we adopted an unbiased method^24,25^ to uncover coherent sets of genes, namely, meta-programs (MPs) that were preferentially co-expressed by subsets of malignant cells based on the scRNA-seq data. In total, we retained seven MPs that recurred in four or more patients (Fig. 2a and Supplementary Table S2). Based on their top-scoring genes, these MPs spanned diverse functions, such as RNA splicing, cell cycle, and ribonucleoprotein biogenesis functions (Fig. 2b and Supplementary Table S3).

MP1 highlighted a subset of cells expressing genes associated with RNA splicing (*SRSF10*, *DDX17*, and *SETX*), indicating that the cells enriched for MP1 (the cells enriched for a specific MP, hereafter referred to as MPX cells, X = 1, 2, …, 7) were in a highly active transcriptional state. Four additional MPs (MP2–MP5) consisted primarily of genes related to immune regulation/response, including humoral immune response (MP2: *XBP1*, *MZB1*, and *IGLC1*), antigen processing, presentation via MHC class II (MP3: *HLA-DRA*, *CD74*, and *B2M*), positive regulation of leukocyte cell‒cell adhesion (MP4: *ICAM1*, *CD83*, and *MAP3K8*), and B-cell-mediated immunity (MP5: *IGHV3-7*, *SERPING1*, and *HLA-DPB1*) (Fig. 2a, b). In addition, MP6 reflected the S and G2/M phases of the cell cycle (Supplementary Fig. S6a–d), representing the proliferative feature of malignant cells in PCNS DLBCL. MP7 was enriched for ribonucleoprotein complex biogenesis (*NPM1/3*, *NHP2*, and *SNRPF*); a gene set enrichment analysis (GSEA) indicated that MP7 cells had elevated telomerase activity (Supplementary Fig. S6e), suggesting that MP7 cells may promote survival by activating telomerase signaling pathways^26^.

To determine potential application of the MPs organization to other GC‐derived B-cell lymphomas, we augmented our data with three publicly available scRNA-seq datasets^13,14,27^. Specifically, these datasets consisted of 11 samples from 9 patients with FL/transformed FL (tFL), 4 samples from 4 patients with ABC DLBCL, and 5 samples from 5 patients with GCB DLBCL (Supplementary Table S4). We embedded the malignant B cells from the above public datasets together with those in our study via UMAP (Fig. 2c), and we obtained an atlas of malignant cells from multiple B-cell lymphomas consisting of 56,966 single cells (Fig. 2d–f), which facilitated the exploration of MPs across these cancers. We used DEPTH2^28^ and general diversity index^29^ to quantify the degree of intratumor heterogeneity of different B-cell lymphomas, which suggested that PCNS DLBCL had considerably higher diversity scores than systemic DLBCL (Supplementary Fig. S7a, b).

Next, we calculated the MP signature scores for each MP in each cell of the integrated dataset (Supplementary Fig. S7c–i; see “Materials and methods”). We then calculated the proportion of MPX cells (X = 1, 2, …, 7) in each sample (Fig. 2g). MP1 cells (5.36% on average) and MP2 cells (6.07% on average) accounted for a small fraction of malignant B cells in PCNS DLBCL. We found that MP1 cells were significantly enriched in PCNS DLBCL over other B-cell lymphomas, suggesting that MP1 is a PCNS DLBCL-specific MP; MP2 cells were significantly enriched in PCNS DLBCL and ABC-like DLBCL patients. MP6 cells reflected the proliferative feature of both PCNS and systemic DLBCL (ABC-like, GCB-like). Taken together, these MPs reflected the unique features of PCNS DLBCL as well as the common features between PCNS DLBCL and systemic DLBCL.

### A PCNS DLBCL-specific phenotype with a tumor-promoting feature

**Fig. 3:**
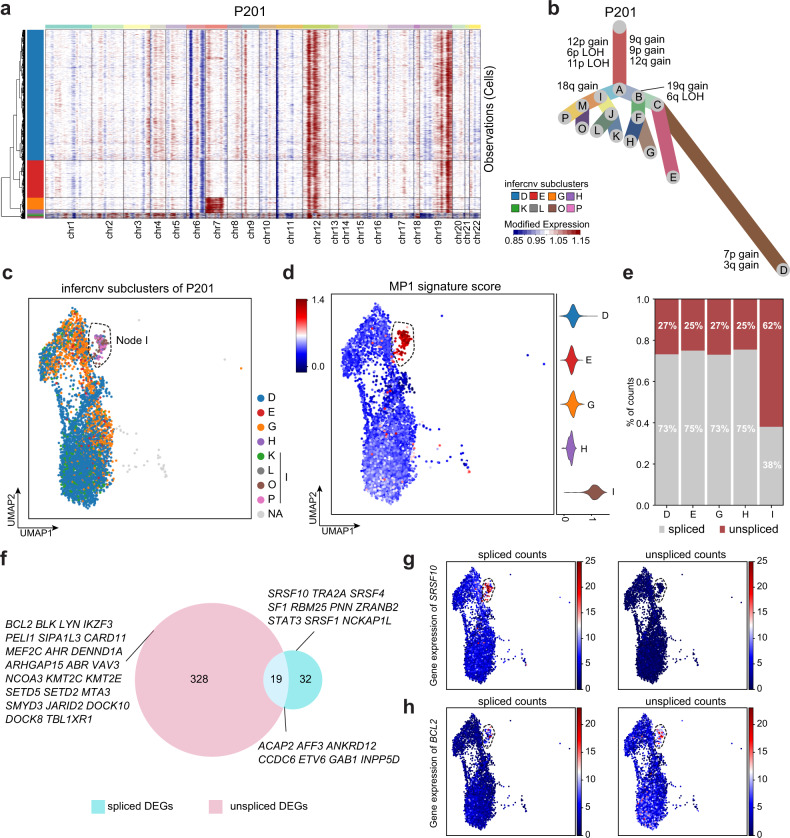
The presence of a PCNS DLBCL-specific phenotype with a tumor-promoting feature. **a** Heatmap of CNV profiles for individual cells (rows) from a representative sample (P201), which were inferred based on the average expression of genes surrounding each chromosomal position (columns). Red: chromosome amplifications; Blue: chromosome deletions. **b** Clonality tree of single cells from sample P201 based on the results of inferCNV. The branches were scaled according to the percentage of cells in the calculated subclone containing the corresponding CNVs. **c** UMAP of malignant cells from P201 colored by nodes of the clonality tree in (**b**). A dashed circle highlights the cells in Node I (including leaf nodes: K, L, O, P). **d** UMAP of malignant cells from P201 colored by the MP1 signature score (left); violin plot showing the MP1 signature score of cells in the nodes of the clonality tree (right). A dashed circle highlights the cells with high MP1 signature scores. **e** Stacked bar plot showing proportions of spliced and unspliced gene counts in cells of clonality tree nodes. **f** Venn diagram showing the DEGs of Node I compared with other nodes only using unspliced and spliced gene counts. Representative DEGs are displayed. UMAP of malignant cells from P201 colored by spliced gene counts (left) and unspliced gene counts (right) for genes *SRSF10* (**g**) and *BCL2* (**h**).

Given that the malignant B cells in PCNS DLBCL were monoclonal or oligoclonal (Supplementary Fig. S4), their intratumor heterogeneity in MPs was less likely to be caused by the origin of the tumor cells. Large-scale chromosomal alterations that occur during tumor progression have been reported to contribute to intratumor heterogeneity^11^. We used inferCNV^20^ to estimate the sample-wise CNV of malignant B cells based on the scRNA-seq data in our study (Fig. 3a and Supplementary Fig. S8). We subsequently used UPhyloplot2^30^ to build clonality trees for each PCNS DLBCL patient based on the inferCNV results (Fig. 3b and Supplementary Fig. S9a). We observed a shallow hierarchy across samples (Supplementary Table S5); taking P201 as a representative example (the number of malignant cells in P201 was 4983), many malignant cells resided in leaf nodes D (69.10%) and E (19.83%), which together constituted node C (88.92%). When we mapped the leaf nodes of the P201 clonality tree into the UMAP embeddings, we found that node I (including leaf nodes K, L, O, and P, consisting of 2.97% of malignant cells in P201) significantly overlapped with the MP1 cells (Fig. 3c, d; *P* < 5.5e−252, hypergeometric test). Interestingly, we observed obvious overlaps between MP1 cells and cells in specific nodes in 6 out of 7 patients (Supplementary Fig. S9b), suggesting that MP1 cells are a common clonal clade of malignant cells in PCNS DLBCL with similar transcriptional features. Moreover, MP1 cells across samples were characterized by two known loci, loss of heterozygosity (LOH) of *HLA-D* locus and 19p13 locus (Supplementary Fig. S10), which have been associated with immune escape and invasiveness^21^. These results suggested that the subclonal CNVs across PCNS DLBCL patients might underlie the formation of transcriptional heterogeneous subpopulations during tumor evolution.

Since genes in MP1 were enriched for genomic functions related to RNA splicing (Fig. 2b), we aimed to uncover whether there were essential genes that promote tumor survival through RNA splicing. We first used velocyto^31^ to quantify the spliced and unspliced counts in cells from P201 and found that cells in node I had a higher proportion of unspliced counts than those in other nodes (62% in node I vs. 26% on average in other nodes; Fig. 3e and Supplementary Fig. S11a). We then conducted differentially expressed gene (DEG) analysis between node I cells vs. cells of other nodes using the spliced or unspliced reads (Fig. 3f and Supplementary Table S6). When only the spliced reads were counted, node I cells had DEGs with known functions related to RNA splicing, such as *SRSF10* (Fig. 3f, g and Supplementary Fig. S11b). When only the unspliced reads were counted, genes encoding protein tyrosine kinases (*LYN* and *BLK*) and Rho GTPases (*ARHGAP15/17/24*) were among the DEGs of node I. These genes were reported to activate B-cell receptor oncogenic signaling^32,33^ and membrane signal transduction^34^, respectively. In terms of unspliced counts, DEGs of node I also showed enrichment for the regulation of B-cell proliferation, in which the antiapoptotic gene *BCL2* was upregulated (Fig. 3h and Supplementary Fig. S11b). Both B-cell receptor signaling and BCL2 have been reported to promote tumor survival and drug resistance in DLBCL^35,36^. Together, these results suggested that cells in node I were capable of hijacking the expression of multiple splicing factors, such as *SRSF1* and *SF1*, apparently leading to dysfunctional gene splicing related to prosurvival pathways and tumor progression.

### Plasmablast-like MP2 cells are associated with a worse prognosis in PCNS DLBCL

**Fig. 4:**
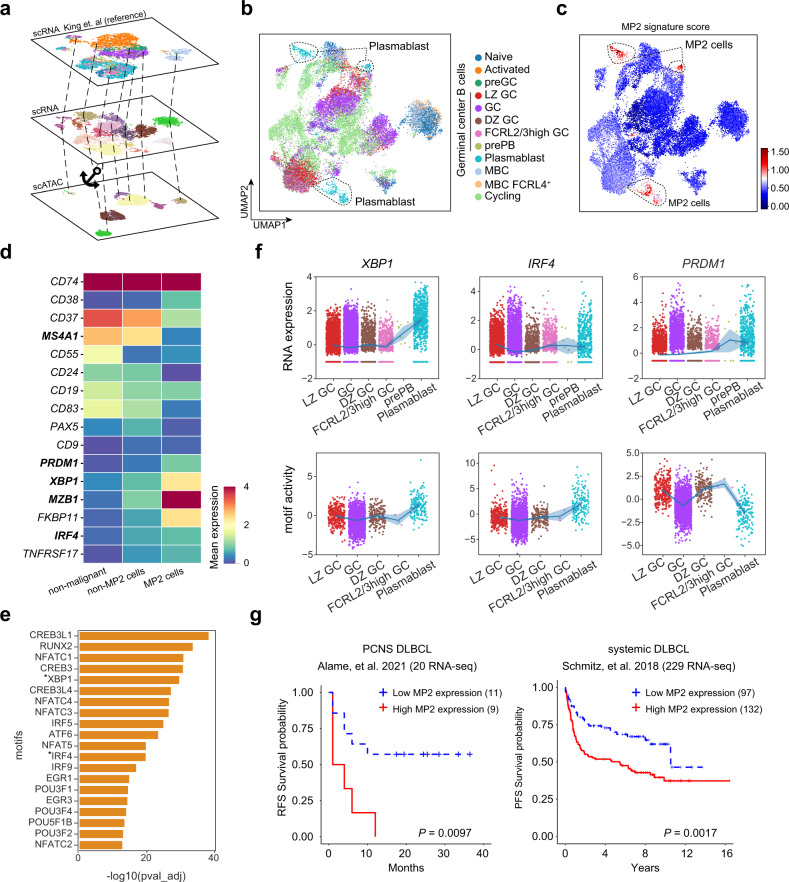
A plasmablast-like expression program in PCNS DLBCL. **a** Schematic of the workflow for joint analysis of scATAC-seq and scRNA-seq data of B cells. We first mapped the PCA embeddings and cluster annotations of the reference dataset (King et al.) to our scRNA-seq data. Then, cluster annotations were transferred from the scRNA-seq data to the scATAC-seq data through canonical correlation analysis. **b** UMAP of B cells colored by GC B-cell cluster annotations transferred from publicly available reference data. Dashed circles highlight the plasmablast cells. **c** UMAP of B cells colored by MP2 signature score. Dashed circles highlight the MP2 cells. **d** Heatmap showing expression levels of selected functional genes in B cells. **e** Bar plot showing the TFs that were significantly enriched in plasmablast-like malignant cells. TFs marked with asterisks are the master TFs for differentiation of plasmablast cells. **f** Strip plot showing the RNA expression levels (upper) and corresponding TF motif activity (bottom) in malignant B cells with GC cluster annotations. **g** The recurrence-free survival (RFS) curve of 20 PCNS DLBCL patients and the progression-free survival (PFS) curve of 229 systemic DLBCL patients based on MP2 signature scores. The *P* values were calculated by the log-rank test.

MP2 was marked by high expression of *MZB1* and *XBP1* (Fig. 2a), which are known marker genes for plasmablast cells, suggesting a plasmablast-like signature in malignant MP2 cells. Therefore, we used publicly available scRNA-seq data from a cohort of normal GC B cells^37^ as a reference to annotate the B cells, including malignant and nonmalignant B cells, from both the scRNA-seq and scATAC-seq data generated in our study (Fig. 4a and Supplementary Fig. S12a–d; see “Materials and methods”). Notably, we detected a strong correspondence between MP2 cells in PCNS DLBCL and the annotated plasmablast cells (Fig. 4b, c; *P* = 0, hypergeometric test). By performing a DEG analysis for MP2 cells vs. other malignant cells and MP2 cells vs. nonmalignant B cells (Supplementary Fig. S12e), we also found a strong plasmablast-like signature in MP2 cells supported by low expression of *MS4A1* and high expression levels of *MZB1*, *XBP1*, and *PRDM1* (Fig. 4d).

We next sought to identify the transcription factors (TFs) that regulate the gene expression program of MP2 cells by investigating the chromatin accessibility of the MP2 cells. We applied chromVAR^38^ and identified 98 TF motifs that were significantly enriched in differentially accessible peaks of MP2 cells compared with other malignant cells (adjusted *P* < 1e−5; Supplementary Table S7). Two out of three master regulators (PRDM1, XBP1, IRF4) that are known to be necessary and sufficient to drive plasma differentiation^39^ were among the top-enriched TFs associated with significant chromatin accessibility in MP2 cells (Fig. 4e and Supplementary Table S7). We then ranked GC clusters from our data according to their annotations, following the normal GC differentiation lineage, and observed that XBP1 and IRF4 were activated while PRDM1 was unactivated in the late GC differentiation stage (MP2 cells) both at the RNA expression and motif activity levels (Fig. 4f), suggesting that the differentiation program of the MP2 B-cell lineage may be partially retained during tumor progression in PCNS DLBCL.

Interestingly, analyses of the independent PCNS DLBCL cohort (*n* = 20)^10^ and systemic DLBCL cohort (*n* = 229)^40^ indicated that patients with a high MP2 signature score showed a significantly worse prognosis than those with a low MP2 signature score (Fig. 4g and Supplementary Fig. S12f).

### Tumor-reactive CD8 T cells are pervasively exhausted in PCNS DLBCL

**Fig. 5:**
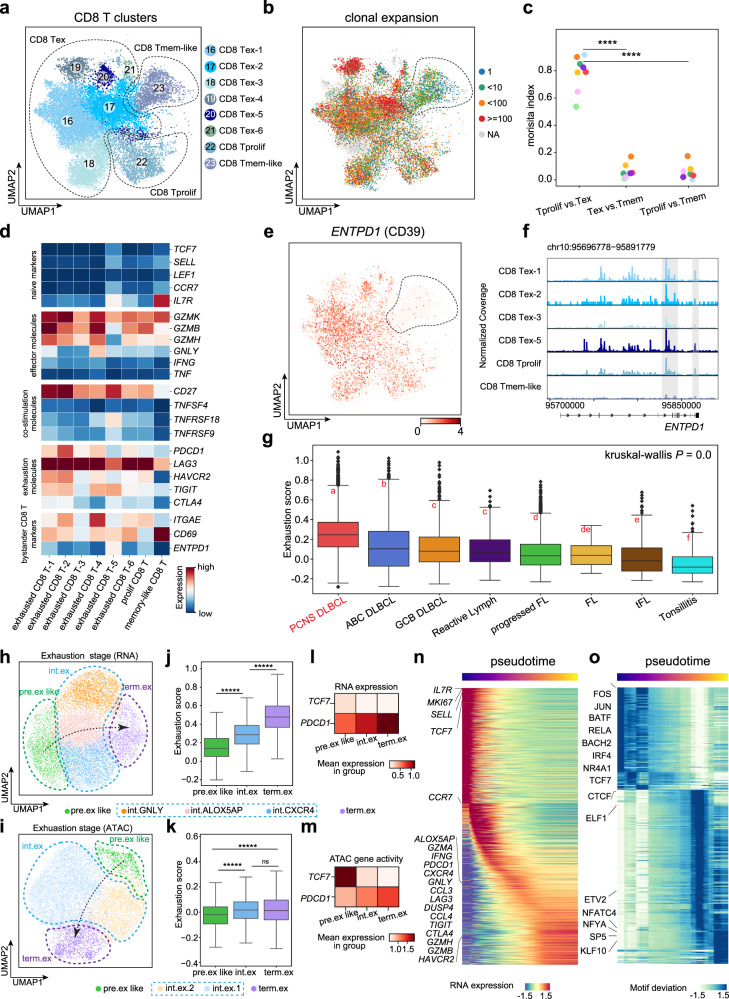
Characterization of exhausted clonally expanded CD8 T cells and bystander CD8 T cells in the TIME. UMAP of CD8 T cells colored by CD8 T clusters (**a**) and clonal expansion of TCR clones (**b**) in the scRNA-seq data. CD8 Tprolif proliferative CD8 T cells, CD8 Tex exhausted CD8 T cells (including exhausted CD8 T-1–6), CD8 Tmem-like memory-like CD8 T cells. **c** Quantification of TCR overlap (Morisita index) between each pair of CD8 T clusters in each patient. Each dot represents a patient. *P* values were calculated by a two-sided Wilcoxon rank-sum test. **d** Heatmap of selected T-cell functional markers for CD8 T-cell clusters. **e** UMAP of CD8 T cells colored by expression levels of gene *ENTPD1* (CD39). **f** Genome tracking plot showing aggregated genomic peaks of *ENTPD1*(CD39) in the scATAC-seq data. **g** Boxplot showing the exhaustion scores of CD8 T cells in different B-cell lymphoma categories. The Kruskal–Wallis test followed by a post hoc test of Fisher’s least significant difference (LSD) was performed to evaluate the significance. Compact letter displays were used to show the significance of the pair-wise comparisons, in which any two groups not sharing any letters were significantly different in exhaustion score. Reactive Lymph Reactive Lymphadenitis. **h** UMAP of tumor-reactive CD8 T cells colored by exhaustion stages identified by trajectory analysis of the scRNA-seq data. **i** is the same as (**h**), but using the scATAC-seq data. **j** Boxplot showing the exhaustion scores of tumor-reactive CD8 T cells in the different exhaustion stages. **k** is the same as (**j**), but using the scATAC-seq data. In (**j**), (**k**), *P* values were calculated by a two-sided Wilcoxon rank-sum test. **l** Heatmap showing expression levels of *TCF7* and *PDCD1* in tumor-reactive CD8 T cells in different exhaustion stages. **m** is the same as (**l**), but using the scATAC-seq data. Heatmap showing the gene expression (**n**) and motif activity (**o**) dynamics along the pseudotime trajectory. In (**g**), (**j**), and (**k**), box boundaries and middle lines correspond to the IQR and median, respectively. Whiskers extend to the lowest or highest data points that are no more than 1.5 times the IQR from the box boundaries.

Distinct dysfunctional states of CD8 T cells as well as bystander CD8 T cells have been observed across human tumors^41–43^. In our study, we identified six clusters of exhausted CD8 T cells (CD8 Tex), one cluster of proliferative CD8 T cells (CD8 Tprolif), and one cluster of memory-like CD8 T cells (CD8 Tmem-like) (Fig. 5a and Supplementary Fig. S2). We then jointly analyzed the clonal expansion and expression of marker genes of these CD8 T cells. The scTCR-seq data showed that the CD8 Tex and CD8 Tprolif cells (25.00%–71.76%, on average 42.16%) had higher proportions of clonal cells than the CD8 Tmem-like cells (16.02%) (Fig. 5b). In addition, CD8 Tex and CD8 Tprolif cells had substantial TCR overlap with each other, while the CD8 Tex and CD8 Tprolif cells showed minimal TCR overlap with the CD8 Tmem-like cells, according to the Morisita-Horn indices, which are widely used to measure TCR overlap between groups^44^ (Fig. 5c and Supplementary Fig. S13a); these trends indicate that CD8 Tmem-like cells have a distinct origin compared to CD8 Tex and CD8 Tprolif cells.

Since the expression of exhausted molecules has been widely used as an indicator of tumor-reactivity T cells in human cancers^45–48^, we then surveyed the expression of selected functional genes in all of the CD8 T cells to evaluate the tumor reactivity of the classified cell clusters (Fig. 5d and Supplementary Fig. S13b). We found that CD8 Tex and CD8 Tprolif cells extensively expressed both exhausted genes (including *PDCD1*, *LAG3*, and *HAVCR2*) and cytotoxic genes (including *GZMK*, *GZMA*, and *GZMB*), while CD8 Tmem-like cells expressed known marker genes of bystander T cells^49^, such as a low level of *ENTPD1* (CD39) and high levels of the tissue-resident marker genes *CD69* and *ITGAE* (CD103) (Fig. 5e, f and Supplementary Fig. S13c, d). Together, these findings suggested that CD8 Tex and CD8 Tprolif cells are tumor-reactive CD8 T cells and that CD8 Tmem-like cells are a cluster of bystander T cells.

Considering that CD8 T cells of PCNS DLBCL pervasively expressed exhaustion molecules (Fig. 5d), we used a predefined exhaustion-related gene signature^50^ to measure the exhaustion level for every single cell. We compared the exhaustion scores of the CD8 T cells in PCNS DLBCL with those that we calculated for tonsil samples and cases including systemic DLBCL, FL, and reactive lymphadenitis^13,14,27^ (Supplementary Fig. S14 and Table S4; see “Materials and methods”). We found significantly higher exhaustion scores in PCNS DLBCL patients than in systemic DLBCL patients (Fig. 5g).

To delineate the exhaustion process of CD8 T cells, we used Monocle3^51^ to infer the developmental stages of the tumor-reactive CD8 T cells from our scRNA-seq data and scATAC-seq data, measured by predicted pseudotime indices (Supplementary Fig. S15a; see “Materials and methods”). We observed a state transition of CD8 T cells from *TCF7*^+^*PDCD1*^+^ pre-exhaustion-like (pre.ex like) to *CCR5*^+^*PDCD1*^+^ intermediate to *HAVCR2*^+^*PDCD1*^+^ terminal exhaustion (term.ex) in both the transcriptomics and chromatin accessibility profiles (Fig. 5h, i), and this finding was supported by the exhaustion scores (Fig. 5j, k), expression trends for selected marker genes (Fig. 5l, m and Supplementary Fig. S15b, c), and the spanning stages of representative TCR clones (Supplementary Fig. S15d).

We then conducted a pseudotime ordering analysis to assess the temporal variability in gene expression and chromatin accessibility during the exhaustion process of tumor-reactive CD8 T cells (Fig. 5n, o). Genes such as *TCF7* and *MKI67* were expressed at the start of the pseudotime trajectory (Fig. 5n), suggesting that the pre-exhausted CD8 T cells have self-renewal capability at an early stage^52^. Inhibitory molecules, such as *PDCD1* and *HAVCR2*, had higher expression levels toward the endpoints (Fig. 5n). Pseudo-ordering of the cells based on motif activity indicated a gradual loss of pre.ex like-specific TF motifs (e.g., AP-1, BATH, BATF, and TCF7) and gain of term.ex-specific TF motifs (e.g., ETS, NF-Y, and KLF; Fig. 5o) along the trajectory.

## Discussion

Relapse and drug resistance are common in patients with PCNS DLBCL and contribute to poor prognosis^53^. The main cause of tumor recurrence and drug resistance lies in intratumor heterogeneity and the complexity of the TIME^11,54^, which is difficult to assess by microarray or bulk sequencing. In the present study, we applied single-cell transcriptome and chromatin accessibility analyses to explore the TIME in PCNS DLBCL patients at single-cell resolution.

Previous bulk genomic studies of PCNS DLBCL identified distinct expression profiles and genomic alterations^15,21^ that distinguished PCNS from systemic DLBCL. In this study, we further characterized the aberrant expression programs of individual PCNS DLBCL patients at single-cell resolution and explored how these programs may contribute to recurrence and drug resistance (Supplementary Fig. S16). For example, we identified a *BCL2*-high phenotype (MP1 cells) specific to PCNS DLBCL that exhibited significant LOH in the *HLA-D* locus, potentially facilitating clonal escape from immune surveillance. These malignant cells displayed elevated expression levels of *LYN* and *BLK*, suggesting the activation of BCR signaling^55^. These findings support the rationale for combining BCR signaling inhibitors with therapeutic interventions for targeting MP1 cells. In addition, we observed the presence of a plasmablast-like program exhibiting a phenotype of *XBP1*^+^*MZB1*^+^*MS4A1*(CD20)^−^. This subpopulation of malignant cells appeared to be unresponsive to the current first-line immunotherapy agent, rituximab, which targets CD20. However, given the plasmacytic phenotype exhibited by these cells, agents such as antimyeloma may offer therapeutic potential^56^. These findings also suggest that a one-size-fits-all treatment may not be effective in PCNS DLBCL. Instead, a personalized or combination targeted therapy approach that considers the unique characteristics of each patient’s tumor heterogeneity may be more effective.

A bulk transcriptomic study previously characterized the TIME of PCNS DLBCL into three immune subtypes according to immune signatures^10^, but was unable to identify the specific composition of different cell types in PCNS DLBCL. In this study, we found that the TIME of PCNS DLBCL was composed mainly of exhausted CD8 T cells and a cluster of bystander CD8 T cells (Supplementary Fig. S16). Interestingly, we also identified a subpopulation of *TCF7*^+^*PDCD1*^+^ CD8 T cells in the TIME of PCNS DLBCL, exhibiting a pre-exhausted-like phenotype. Recent studies have demonstrated that pre-exhausted CD8 T cells have the capacity to proliferate and exert antitumor activity upon anti-PD-1 treatment in patients with lung cancer^57^. Furthermore, unraveling the molecular regulatory mechanisms underlying the development of pre-exhausted CD8 T cells has been highlighted as a key strategy for reversing T-cell exhaustion^58^. Therefore, this population of pre-exhausted-like CD8 T cells may represent a promising target for immune checkpoint therapy, and the regulatory programs observed in our study may facilitate the identification of critical molecules involved in reversing T-cell exhaustion.

We produced high-quality single-cell profiles and conducted unbiased analyses to reach our findings, yet there are limitations in this study. A limitation of our analyses is its limited samples, which might be not sufficient to fully elucidate the heterogeneous gene programs in PCNS DLBCL. Among the discovered meta programs, MP1 is related to an aberrant splicing signature, and comprehensively confirming its mechanism remains a challenge. Moreover, we applied computational strategies to integrate scRNA-seq data and scATAC-seq data, which remained an analytical challenge to the field. Cutting-edge methods enabling profiling of chromatin accessibility and transcriptome within the same single cell could be utilized to precisely dissect underlying mechanisms that drive the expression programs. Nonetheless, our high-throughput and multi-omics profiling of PCNS DLBCL as well as our follow-up analyses of independent cohorts facilitated the understanding of both intratumor heterogeneity and TIME complexity of PCNS DLBCL, which could help promote the development of targeted therapies in this malignancy.

## Materials and methods

### Patient samples and tumor tissue processing

This study was approved by the ethics committee of The First Affiliated Hospital of the University of Science and Technology of China (No. 2022-KY-091). Informed consent was obtained in advance. The study was compliant with all of the relevant ethical regulations regarding research involving human participants. Eight patients who underwent diagnosis for PCNS DLBCL tumors at The First Affiliated Hospital of USTC were evaluated. All samples were obtained as surgical biopsies and mechanically dissociated into single-cell suspensions. The scRNA-seq and scATAC-seq were performed using both fresh and frozen samples (Supplementary Table S1). To prepare frozen samples, we placed fresh cells in 90% FBS (Gibco) supplemented with 10% DMSO and then cryopreserved them in liquid nitrogen.

### Flow cytometry gating

Cells were stained with CD19-APC, CD45-APC-Cy7, DAPI, and Calcian for measurement by flow cytometry. Due to the various proportions of malignant cells in cell suspensions, we set up different gating methods for different tissues to manipulate the proportion of malignant cells to nonmalignant cells (Supplementary Table S1). After enough cells were sorted, they were spun down and resuspended in 0.04% BSA (dissolved in 1× PBS). Trypan blue dye staining was performed to determine quality and quantity of cells by an Invitrogen Countess II device. Then, the cell suspension was diluted to an appropriate concentration before library preparation for scRNA-seq or nuclei isolation for scATAC-seq.

### Library preparation and sequencing of scRNA-seq paired with BCR-seq and TCR-seq

Single-cell libraries were generated with the 10× Genomics Chromium Single Cell 5′ (v1.0, *n* = 8) and V(D)J (v1.0, *n* = 7) assays (Supplementary Table S1) before sequencing on the Illumina NovaSeq 6000 instrument with 150/8/150-bp (scRNA and scVDJ) read configurations.

### Nuclei isolation, library preparation, and sequencing of scATAC-seq

After sorting, we isolated, washed, and counted the nuclei suspensions according to the demonstrated protocol from 10× Genomics. Nuclei were spun down at 500× *g* for 5 min at 4 °C and resuspended in diluted nuclei buffer. Then, we proceeded immediately to scATAC-seq library construction using Chromium Single-Cell ATAC Solution v1.1 kit (10× Genomics). Check size distribution of libraries using an Agilent 2100 bioanalyzer before sequencing. scATAC-seq libraries (*n* = 5) were sequenced on a NovaSeq 6000 (Illumina) instrument with 50-bp paired-end reads.

### Quality control of scRNA-seq data

We collected a total of nine samples from eight PCNS DLBCL patients for scRNA library preparation and sequencing, and processed the FASTQ files with typical workflow of CellRanger (v5.0.1) software to obtain gene expression count matrix of each sample. Scanpy (V1.8.2)^18^ software was used to merge the raw count matrix of each sample and subsequently conduct a quality control analysis. For gene filtering, genes that were expressed in less than 50 cells were removed. For cell filtering, cells were selected with the following principles: (1) the number of expressed genes was from 500 to 6000, (2) the mitochondrial RNA content was lower than 15%, and (3) the total counts of each cell were less than 50,000. Then, DoubletDetection software (https://github.com/JonathanShor/DoubletDetection) was used to detect potential doublets in each sample (n_top_var_genes = 2000, boost_rate = 0.5, voter_thresh = 0.9). Notably, for patient P201, we observed potential contamination and used Gaussian mixture models to identify and remove cells that expressed multiple canonical markers across cell types; for patient P203, we also removed a cluster of cells co-expressing multiple canonical markers across cell types (co-expressing *CD68*, *CD3D*, *CD19*). Finally, a total of 49,910 single-cell transcriptomes were retained after quality control.

### Quality control of scATAC-seq data

We performed scATAC library preparation and sequencing on 5 of the samples that were processed for scRNA sequencing. We first used typical workflow of CellRanger ATAC (v1.2.0) software to preprocess the samples, including alignment of raw reads to the hg38 human genome, peak calling for each sample, and combining the outputs of each sample to obtain a unified peak-barcode matrix. Then, we used Signac^59^ to perform quality control following the standard workflow (cells with a TSS enrichment score less than 2.5 were filtered out). Moreover, scDblFinder^60^ was applied to detect and remove potential doublets following the typical tutorial for scATAC-seq data. After quality control, a total of 31,833 chromatin profiles were retained.

### Dimensionality reduction and clustering of scRNA-seq data and scATAC-seq data

For scRNA-seq data, we first normalized the gene expression of each cell to 10,000 and performed a logarithmic analysis. After that, we selected the top 2000 most variable genes for subsequent dimensionality reduction and clustering analysis. We conducted principal component analysis (PCA) on the gene expression matrix and used the first 40 principal components (PCs) for UMAP. In the first round of clustering, major cell types, including NK&T cells, B cells, myeloid cells, and oligodendrocytes, were identified by Louvain clustering with a resolution of 0.05 and merged based on canonical markers (*MS4A1* for B cells; *CD3D* for T cells; *TYROBP* for NK cells; *CD163* for myeloid cells; and *MOG* for oligodendrocytes). Next, we performed a second round of clustering to further characterize subpopulations of NK&T and B-cell types. Owing to the variable amount and property of cells in each major cell type, different parameters for clustering were used. For the clustering of NK&T cells, the top 40 PCs were selected on the basis of 2000 highly variable genes (HVGs) (resolution = 0.8). For the clustering of B cells, the top 40 PCs were selected on the basis of 2000 HVGs (resolution = 0.5). As a result, we identified NK cells (*TYROBP*), 8 CD8^+^ T subpopulations (*LAG3*, *PDCD1*, and *HAVCR2* for exhausted CD8 T; *MKI67* for proliferative CD8 T, *GZMK*, *IL7R* for memory-like CD8 T), 2 CD4^+^ T subpopulations (*GZMB* for cytotoxic CD4 T, *CD27* for activated CD4 T), Treg (*FOXP3*) and gdT (*TRDV2*) for T-cell type based on canonical markers and DEGs. We also identified 16 B-cell clusters for B-cell type; the majority of B cells strongly clustered according to the patient of origin. This phenomenon was also observed in the Smart-seq data of PCNS DLBCL^15^ and single-cell data of other human cancers^61–63^. Cluster B14 was filtered out in the subsequent analysis because it was likely to be a cluster of doublets (Supplementary Fig. S2b). Notably, in the second round of clustering, we applied the harmony algorithm^64^ to remove the potential batch effect among samples.

For the scATAC-seq data, we used Signac’s typical workflow to analyze the chromatin profiles. We normalized the cell-peak matrix by using the term frequency-inverse document frequency (TF-IDF) method. Then, we selected the top 15% of highly variable features for downstream dimensionality reduction and clustering. We retained a reduced dimension representation of the scATAC-seq data by running singular value decomposition on the TF-IDF matrix. UMAP was then calculated for data visualization. We conducted a smart local moving algorithm to cluster the chromatin profiles, yielding 23 clusters of cells. To annotate the major cell types by canonical markers (*MS4A1* for B cells; *CD3D* and *CD8A* for CD8 T cells; *CD3D* and *CD4* for CD4 T cells; *TYROBP* for NK cells; *CD163* for myeloid cells; and *MOG* for oligodendrocytes), gene activity scores of each gene, which could be used as a proxy for gene expression, were calculated by summing the fragments intersecting with the region of gene body and 2 kb upstream of the transcription start site. Then, we used the scRNA-seq data as a reference and mapped cluster annotations from the scRNA-seq data to the scATAC-seq data^19^. Specifically, we aligned the gene activity scores and gene expression matrix into a shared low-dimensional space by using the canonical correlation analysis (CCA) algorithm. We then identified anchors between the scRNA-seq data and scATAC-seq data. The anchors are pairs of cells from each dataset that are contained within each other’s neighborhoods, which were finally used to transfer cluster annotations from scRNA-seq data to scATAC-seq data. Genome track plots of representative regions or genes were generated by using ArchR^65^.

### scBCR-seq and scTCR-seq data analyses

Raw sequencing FASTQ files of BCR and TCR libraries were analyzed using CellRanger (v5.0.1). The CellRanger outputs of each sample contained an output “filtered_contig_annotations.csv”, which was used in the downstream analysis. We then sequentially intersected the filtered contig annotations of each sample with coarse transcriptomic cell types (T cells and B cells) using Scirpy^66^. Following the typical Scirpy analysis workflow, we filtered out the cells that contained multiple chains and defined the clonotypes based on the nucleotide sequence of complementary-determining region 3. We calculated the proportions of clonal cells among CD8 T-cell clusters by counting the proportion of clonally expanded cells with a clone size of more than 10. Moreover, the clonal overlap between each pair of CD8 T clusters was calculated using the Morisita index implementation of immunarch (https://github.com/immunomind/immunarch), and the Berger-Parker index was used to represent the dominant score of clonal expansion with function alpha.berger_parker_d in skbio (https://github.com/biocore/scikit-bio).

### Identification of malignant B cells

We used two different strategies to identify malignant B cells. First, we used inferCNV (V1.10.1)^20^ to estimate the chromosomal CNVs following the standard workflow for 10× genomics with default parameters. Since we aimed to distinguish malignant B cells from nonmalignant B cells, we used all nonmalignant B cells as reference cells. Then, to confirm the inferCNV results, we exploited the phenotypic allelic exclusion of malignant B cells and calculated the relative expression of kappa and lambda chains for each cell. Accounting for the differential gene expression level in various kappa or lambda genes, both kappa genes and lambda genes with the maximum gene expression level in each cell were selected for the calculation of relative expression.

### COO classification

We applied a previously reported ABC and GCB classification method to each malignant B-cell^23^. Specifically, we retained the genes previously reported to mark ABC and GCB subtype^67^, then performed quantile normalization and log_2_ transformation on gene expression measurements of this gene in all malignant cells, followed by z-normalization across these genes. Then ABC and GCB scores were computed for each malignant cell by taking the average of *z*-scores for ABC and GCB genes, respectively. A combined subtype score was then computed by taking the difference between ABC score and GCB score. A malignant cell was classified as ABC if the combined subtype score was > 0.25 and its GCB score was < 0.75; and it was classified as GCB if the combined subtype score was < –0.25 and its ABC score < 0.75. The rest of the malignant cells belonged to the unclassified group.

### Single-cell meta-program analysis

We performed a consensus nonnegative matrix factorization (cNMF) analysis on malignant cells from each sample by using cNMF (v1.2)^68^ so that we retained modules for each sample. Notably, cluster B14 was filtered before cNMF analysis because it was likely to be a cluster of doublets (Supplementary Fig. S2b). Then, we used Scanpy^18^ to calculate the gene scores of each module. The gene scores of single cells for each module were also used to manually select different thresholds to distinguish specific module-enriched cells from nonenriched cells. Next, we calculated the pair-wise Pearson correlations within all modules and obtained a correlation matrix. Finally, we performed hierarchical clustering on the correlation matrix to compartmentalize the meta-programs.

### Gene signature score analysis

Gene signature score analysis is widely used to quantify the average gene expression level for a given gene set compared to a randomly selected reference gene set. In this study, we used the function *score_genes* in Scanpy^18^ with default parameters to quantify the activity of gene sets derived from MP analysis, cell cycle state gene sets, and exhaustion gene sets for CD8 T cells. The gene sets for evaluating the cell cycle states were extracted from the previously reported scoring system^69^.

### Differential expression analysis

In this study, we performed DEG analysis to identify the DEGs between two groups of cells by using the Wilcoxon rank-sum test. To identify the DEGs between *MZB1*^+^ and *MZB1*^−^ or nonmalignant B cells, we first categorized the malignant B cells into *MZB1*^+^ and *MZB1*^–^ cells with a threshold of 0.9 (MP2 signature score). To calculate the DEGs of node I against other branches in P201 in terms of spliced and unspliced counts, we first quantified the unspliced counts, spliced counts^31^ and conducted DEG analysis by using the Wilcoxon rank-sum test with a threshold: adjusted *P* value ≤ 0.01, fold change ≥1.5 (Fig. 3f). The *P* value was corrected by using the Benjamini–Hochberg method.

### Clonal evolution analysis

To construct the evolution tree diagram, we used inferCNV^20^ to detect sample-wise CNVs of malignant B cells compared with nonmalignant cells. Cell clusters with less than 20 cells in each sample were removed to filter out the potential noise raised by cell clustering. We ran inferCNV using the default parameters except setting tumor_subcluster_partition_method and analysis_mode to random_trees and subclusters. Then, with CNV analysis of each sample, the Python package UPhyloplot2^30^ was used to draw a tumor cell evolution tree diagram to study the evolution of tumor B cells in each sample. We individually utilized the R package CopyscAT^70^ to infer CNV data from the scATAC-seq data for each sample. After obtaining the results of unsupervised clustering for CNV data identified by NMF, we manually annotated the nonmalignant cell clusters and used them as controls to call CNVs in the malignant cell clusters.

### Cell type mapping of external scRNA-seq data

We used the *ingest* function in Scanpy^18^ to project the cell annotations from the published scRNA-seq data of normal tonsils^71^ onto our data based on the PCA embeddings to prove that MP2 cells closely resembled a cluster of plasmablast-like cells.

### Trajectory inference of CD8 T cells from the scRNA-seq data

Since bystander T cells were supposed not to be associated with tumorigenesis and progression, we performed trajectory inference on tumor-specific CD8 T cells (exhausted CD8 T-1, exhausted CD8 T-2, exhausted CD8 T-3, exhausted CD8 T-4, exhausted CD8 T-5, exhausted CD8 T-6 and prolif CD8 T cells). First, a Gaussian mixture model was applied to remove the potentially contaminated cells based on the expression levels of gene *MS4A1*. Then, we borrowed the methods from Zheng et al.^42^ to define a gene blacklist and disassociation-induced gene (DIG) signature. The gene blacklist contained genes located on the X and Y chromosomes, immunoglobulin genes, and T-cell receptor genes from the R package biomaRt, ribosome-protein-coding genes, *MALAT1*, and marker genes of the exhausted CD8 T-3 cluster, which are associated with tissue dissociation operations, including heat shock protein-encoding genes^72^. We excluded genes from the blacklist and identified the top 2000 genes as HVGs. Then, the unwanted effect caused by the DIG signature, cell cycle, percentage of mitochondrial UMI counts, and total UMI counts were regressed out before performing PCA. The donor effect was removed by Harmony^64^. Then, the harmony embeddings were used to build the neighborhood graph for cell clustering and UMAP. Then, Monocle3^51^ was adopted to introduce pseudotime and build trajectories based on the cell clusters and UMAP embeddings. The cell exhibiting the highest expression level of *TCF7* was set as the root cell, and the pseudotime was calculated by the function order_cells. We divided the cells into 100 portions according to the pseudotime and calculated a pseudobulk expression matrix. Identification of the top variable features across the trajectory was performed using ArchR’s plotTrajectoryHeatmap function based on the pseudobulk expression matrix.

### Trajectory inference of CD8 T cells from the scATAC-seq data

Based on the mapped cluster annotations based on the scRNA-seq data, we selected tumor-specific CD8 T cells (exhausted CD8 T-1, exhausted CD8 T-2, exhausted CD8 T-3, exhausted CD8 T-5, and prolif CD8 T cells) for trajectory analysis. First, we used Signac’s FindIntegrationAnchors function to find integration anchors between the five patients’ cells based on the previously calculated LSI embeddings. Then, Signac’s IntegrateEmbeddings function was used to calculate the integrated LSI embeddings. The RunUMAP, FindNeighbors, and FindClusters functions were performed using the integrated embeddings. Gene scores were calculated using ArchR’s addGeneScoreMatrix function, and gene score imputation was performed with Magic using ArchR’s addImputeWeights function^73^. Then, we adopted Monocle3^51^ to build the trajectory. The cluster with the highest score for the *TCF7* gene was specified as the root, and then the pseudotime was calculated using the function order_cells. The enrichment of motif deviation was performed with ChromVAR^38^ using ArchR’s addDeviationsMatrix function. We added the trajectory built by Monocle3 to the ArchR project and then used ArchR’s functions getTrajectory and plotTrajectoryHeatmap to identify top variable motif deviations across the trajectory.

### Survival analysis

To confirm whether MP2 was associated with clinical outcomes, we conducted a survival analysis. Information for twenty PCNS DLBCL patients and 234 systemic DLCBL patients with complete follow-up records was downloaded from GEO (GSE155398)^10^ and NCICCR DLBCL^40^, respectively. All systemic DLBCL and PCNS DLBCL patients had records of overall survival. Moreover, 20 PCNS DLBCL patients had records of RFS, and 229 of 234 systemic DLCBL patients had records of PFS. We first calculated the single-sample GSEA signature score of MP2 by using the function calculate_sig_score in the R package IOBR^74,75^ and then splited the patients into a high-expression group and a low-expression group with the optimal cutpoint determined by the surv_cutpoint function in the R package survminer (https://github.com/kassambara/survminer) with the parameter minprop = 0.3. Finally, we employed the survfit function in the R package survival (https://github.com/therneau/survival) to evaluate the impact of MP2 on the clinical outcomes (parameters: type = Kaplan‒Meier, error = tsiatis).

### Motif enrichment analysis

We observed a subset of malignant B cells showing the characteristics of plasmablast signatures in normal B cells. We aimed to dissect the potential regulators underlying this subset of malignant B cells. First, we applied the CCA algorithm implemented in Seurat^19^ to transfer the GC cluster annotations from the scRNA-seq data to the scATAC data. Then, we recalled peaks independent of the groups of GC annotations and then combined them by using the CallPeaks function in Signac^59^. In addition, we conducted a differential motif activity analysis on a per-cell motif activity score obtained from chromVAR analysis^38^. We also used known motifs in the JASPAR database (JASPAR2020) to find overrepresented motifs that were enriched in plasmablast-like malignant B cells. Finally, the top 20 overlapping motifs between overrepresented motifs and differential activity motifs were shown.

### Integrative analysis with published scRNA-seq data

To determine whether the subset of plasmablast-like malignant B cells is also present in other GC-derived B lymphomas, we conducted an integrative analysis with malignant B cells of systemic DLBCL and FL patients in three other independent cohorts^13,14,27^ that were subjected to scRNA-seq using the routine scRNA-seq analysis workflows of Scanpy with default parameters. Malignant cells in each dataset were extracted based on the cluster annotations in the original studies. Moreover, to compare the exhaustion degree of CD8 T cells between PCNS DLBCL and extracerebral B lymphomas, we also conducted an integrative analysis with CD8 T cells from the same datasets as above using the same analysis process. We extracted CD8 T cells based on the cluster annotations in the original studies. After the integrative process, the T-cell exhaustion signature was used to calculate the exhaustion score.

### Comparison of intratumor heterogeneity

We employed two methods to quantify the degree of intratumor heterogeneity (ITH) in PCNS DLBCL and compare it with those in other B-cell lymphomas. Firstly, we quantified the tumor’s ITH level based on the standard deviations of absolute *z*-scored expression values of genes by using the DEPTH2 algorithm^28^. We calculated the mean expression of each gene across all cells to form a pseudo-bulk RNA-seq data as the input of DEPTH2. We also applied the general diversity index^29^ to quantify the ITH degree at the single-cell level. Specifically, after normalization and dimensionality reduction, all malignant cells were clustered into different clusters by the unsupervised Louvain algorithm with default parameters. Then, the diversity index was calculated using the cellular frequencies over clusters across a range of the order of diversity *q* values. Different *q* values correspond to different meanings: Species (clonal) richness of a sample is given by *q* = 0. The Shannon index (log scale) can be found when q approaches 1. The Simpson index, which approximates the probability that any two cells are identical, emerges from the case of *q* = 2.

### Signature enrichment analysis

GSEA was performed by using GSEApy (https://github.com/zqfang/GSEApy) with gene sets from the Molecular Signatures Database (MSigDB). Gene Ontology (GO) enrichment analysis in this study was performed by using clusterProfiler (V4.2.2)^76^.

### Statistics

DEG analysis in this study was performed by using the Wilcoxon rank-sum test. In Fig. 5g, the Kruskal‒Wallis test followed by a post hoc test of the criterium Fisher’s LSD was performed for *P* value calculation using the kruskal function in the R package agricolae (https://github.com/cran/agricolae). Other statistical methods and tests used in this paper are described in the corresponding figure legends.

## Supplementary information

Supplementary Information

STable 1

STable 2

STable 3

STable 4

STable 5

STable 6

STable 7

## Data Availability

All the raw data of scRNA-seq, scBCR-seq, scTCR-seq and scATAC-seq have been deposited in the Genome Sequence Archive (GSA) for Human in the BIG Data Center (https://ngdc.cncb.ac.cn/gsa-human/), under the accession number HRA002297. The corresponding processed data are available at the Zenodo data archive (https://zenodo.org/record/7813151).
