# Supplementary material for: Single-cell landscape of primary central nervous system diffuse large B-cell lymphoma
Source: Cell Discov. 2023 Jun 12;9:55. doi: 10.1038/s41421-023-00559-7 (PMC10261103; doi:10.1038/s41421-023-00559-7)
Supplement: Supplementary file 1 — Supplementary Information [file 41421_2023_559_MOESM1_ESM.pdf]

Supplementary information

This file includes: Supplementary Fig. S1 to S16

Supplementary Fig. S1

a

Clinical information

Patient	IHC	ISH	HIV	scATAC
P73	CD45^{+}, Pax-5^{+}, CD20^{+}, Bcl-2^{+}, Bcl-6^{+}, MUM-1^{+}, CD10^{-}, c-Myc $^{+}$, GFAP $^{-}$, CD3 $^{-}$, CD5 $^{-}$, KI-67^{+}(90%)	EBER-ISH -	negative	Yes
P124	Pax-5^{+}, CD10^{+}, MUM-1^{+}(weak), Bcl-6^{+}, Bcl-2^{+}, c-Myc^{+}(20%), CD30^{-}, CD5^{-}, CD3^{-}, Cyclin-D1^{-}, pan-CK^{-} , Syn^{-} , KI-67^{+}(85%), CD20^{+}	EBER-ISH -	negative	No
P145	Pax-5^{+}, CD10^{+}(weak), MUM-1^{+}(weak), Bcl-6^{+}, Bcl-2^{+}(weak), c-Myc^{+}(20%), GFAP^{-}, CD3^{-}, CD5^{-}, p53^{+}(5%), KI-67^{+}(80%), CD20^{+}	EBER-ISH -	negative	Yes
P182	Pax-5^{+}, Bcl-2^{+}, Bcl-6^{+}, CD10^{-}, MUM-1^{+}(weak), c-Myc^{-}, p53^{+}, CD3^{-}, CD5^{-}, CD15^{-}, CD30^{+}, GFAP(brain)$^{+}$, KI-67^{+}(90%), CD20^{+}	EBER-ISH -	negative	No
P201	CD20^{+}, Pax-5^{+}, CD3^{-}, CD5^{-}, Bcl-6^{+}(70%), CD10^{-}, MUM-1^{+}(80-90%), Bcl-2^{+}(>90%), CD30^{-}, p53^{+}(80%), c-Myc^{+}(60-70%), cyclin-D1^{-}, KI-67^{+}(90%)	EBER-ISH -	negative	Yes
P202	CD20^{+}, Pax-5^{+}, CD10^{-}, MUM-1^{+}(60%), Bcl-6^{+}(70%), Bcl-2^{+}(80%), CD30^{-},CD3^{-}, c-Myc^{+}(weak), p53^{+}(70%), Cyclin-D1^{-}, SOX11^{-}, CD5^{-}, pan-CK^{-}, KI-67^{+}(80%)	EBER-ISH -	negative	Yes
P203	CD20^{+}, MUM-1(weak), Bcl-6(weak), CD10^{-}, CD21^{+}(weak), Bcl-2^{+}(85%), c-Myc^{+}(60%), p53^{+}(80%), GFAP^{+}, Olig2^{+}, CD3^{+}(weak), pan-CK^{-}, KI-67^{+}(90%)	EBER-ISH -	negative	No
P205	GFAP^{+}(weak), Desmin^{+}(weak), WT1^{+}(weak), TdT^{+}(weak), INI1^{+}, BRG1^{+}, Myogenin^{-}, MyoD1^{-},BCoR^{-}, Olig2^{+}(weak), NeuN^{-}, G34R^{-}, CD3^{-}, CD20^{-}, Pax-5^{-}, CD99^{-}, CD34^{-}, CD33^{-},CD7^{-}, CD5^{-}, MPO^{-}, Cyclin-D1^{-}, CD43^{-}, LCA^{-}, CD38^{-}, CD79a^{-}, S100^{-}, Syn^{-}, pan-CK^{-}, SALL4^{-}, KI-67^{+}(50%)	EBER-ISH -	negative	Yes

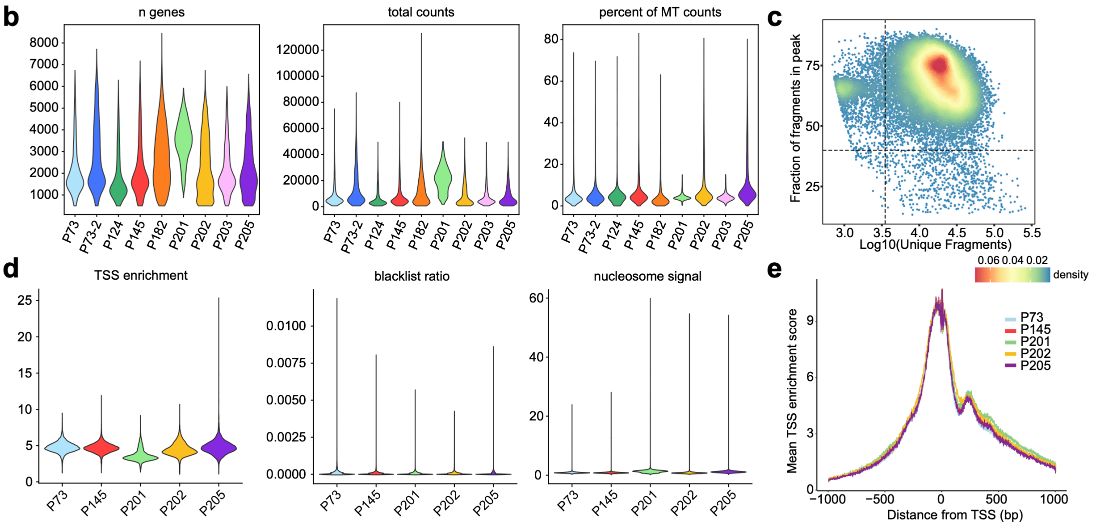

Supplementary Fig. S1 Clinical information and quality control of scRNA and scATAC data. a Table summarizing the clinical information of patients, including the reports of Immunohistochemistry (IHC), in-situ hybridization (ISH) of Epstein-Barr virus small RNA (EBER), HIV infection status, and the patients with sufficient cells for scATAC-seq library preparation (all samples were subjected to scRNA-seq experiments). **b** Violin plot of detected genes (left), total counts (middle), and percentage of mitochondria (MT) counts (right) in scRNA-seq data. **c** Density scatter plot showing the fraction of fragments in peak vs log10(unique fragments per cell), colored by density. The horizontal and vertical dashed lines represented the thresholds of quality control in

scATAC-seq data. **d** Violin plot showing transcriptional start site (TSS) enrichment (left), blacklist ratio (middle), and nucleosome signal (right) in scATAC data. Dashed lines indicated the quality control threshold. **e** TSS enrichment of each patient in scATAC-seq data.

Supplementary Fig. S2

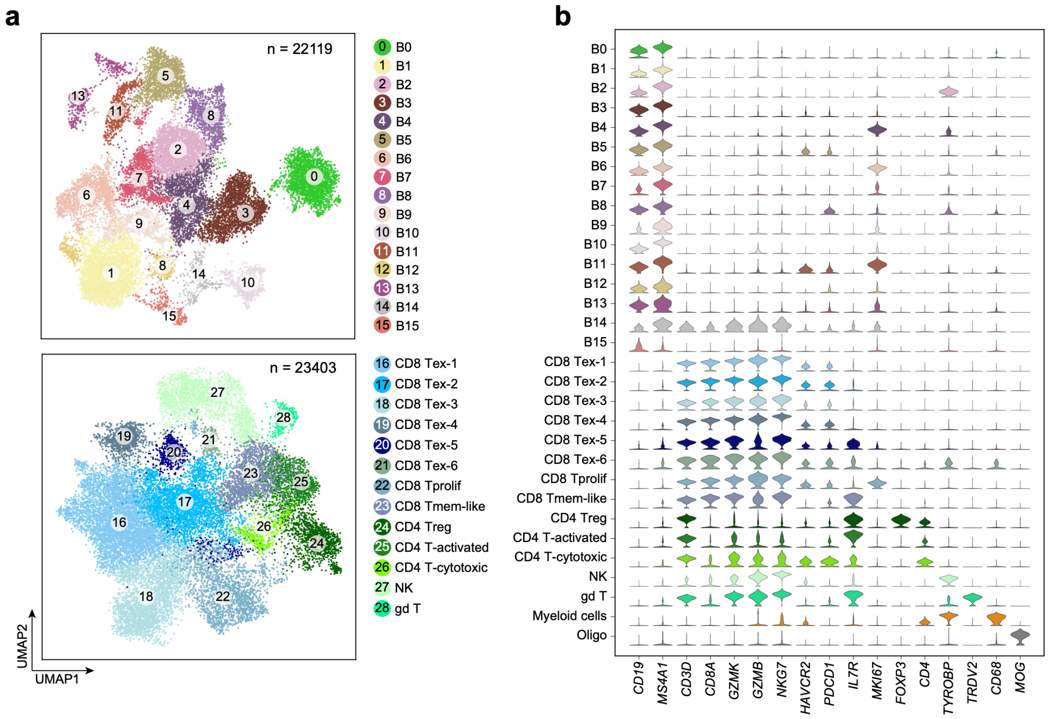

Supplementary Fig. S2 Cell type characterization of scRNA-seq data. a UMAP of B cells (upper) and NK&T (bottom) cells from scRNA-seq colored by cell clusters. **b** Violin plots showing the expression of canonical marker genes for scRNA-seq cell clusters.

Supplementary Fig. S3

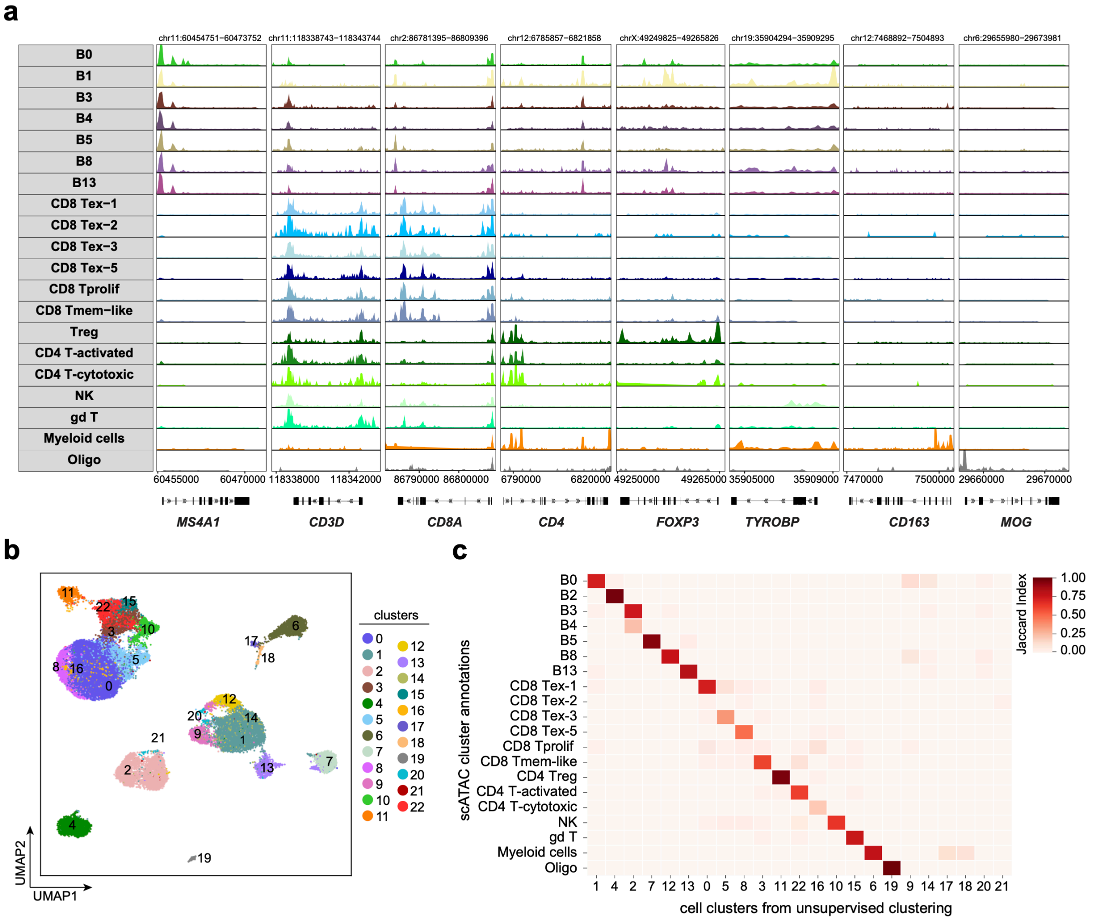

Supplementary Fig. S3 Cell type characterization of scATAC-seq data. a Genome tracking plots showing aggregated genomic peaks of canonical marker for cell clusters in scATAC-seq data. **b** UMAP of all cells colored by cell clusters yield from unsupervised clustering in scATAC data. **c** Heatmap of Jaccard index showing the pairwise correspondence between unsupervised cell clusters and label-transferred clusters.

Supplementary Fig. S4

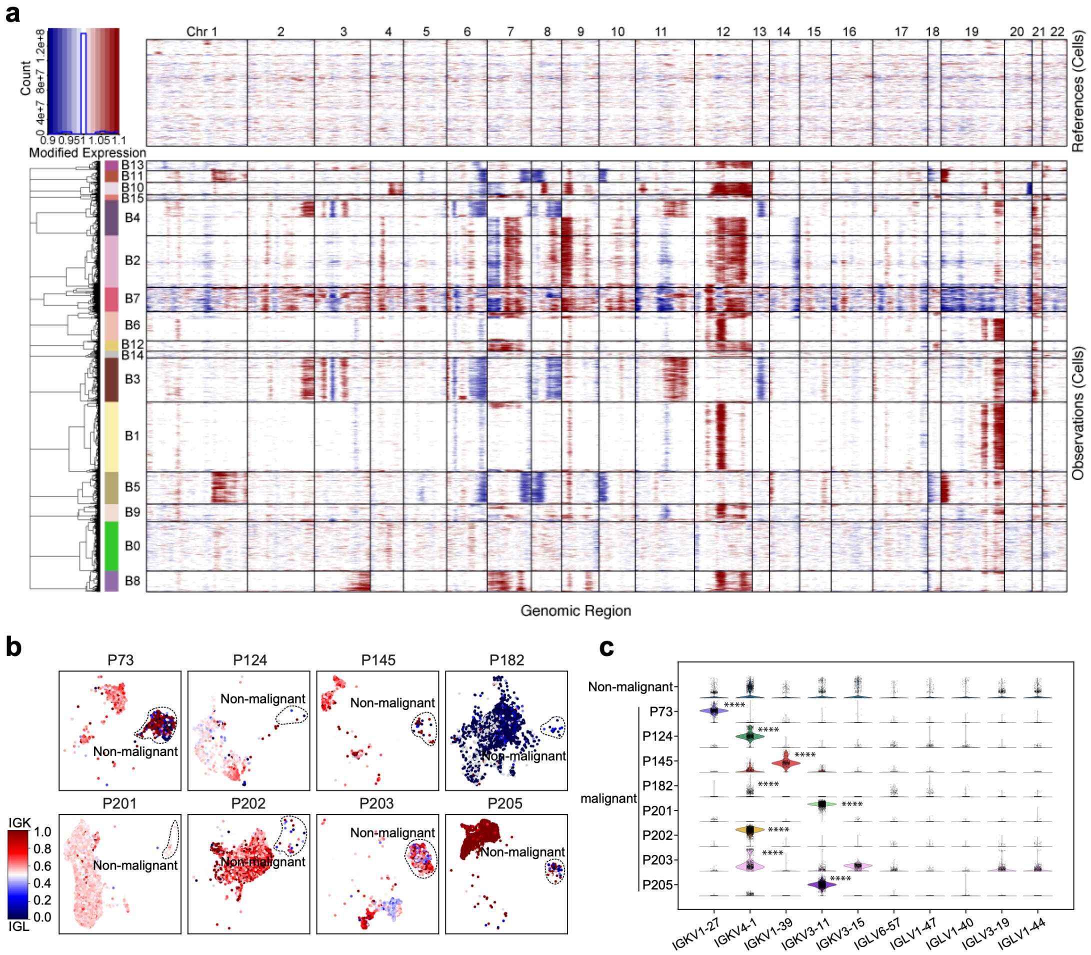

Supplementary Fig. S4 Identification of malignant B cells. a Heatmap showing CNVs for individual B cells (rows) from all samples, inferred based on the average expression of genes surrounding each chromosomal position (columns). Red: amplifications; blue: deletions. **b** UMAP of B cells in each patient colored by the relative expression of IGK genes to IGL genes. Non-malignant cells were highlighted by dashed circle. **c** Violin plot showing the expression of dominantly expressed BCR light chain genes in malignant cells of each patient and non-malignant cells of all patients. A two-sided Wilcoxon rank-sum test was performed between the dominantly expressed light chain and other light chains (***P < 0.001, **P < 0.01, *P < 0.05).

Supplementary Fig. S5

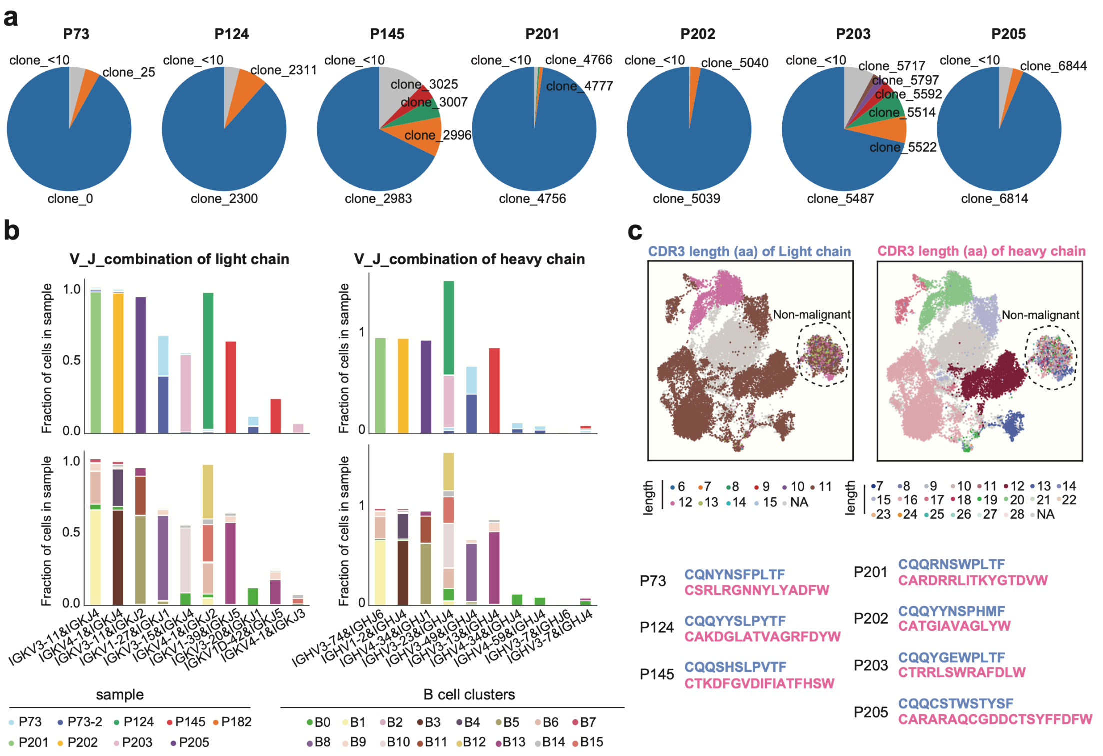

Supplementary Fig. S5 scBCR-seq data analysis. a Pie plot showing the proportion of BCR clones in each patient. BCR clones less than 10 were aggregated to calculate the proportion. **b** Stacked barplot showing the distribution of VJ combination in light (left) and heavy (right) chain, colored by sample (upper) and B cell clusters (bottom) respectively. **c** UMAP of B cells colored by BCR complementarity-determining region (CDR3) amino acid sequence length of light (left) and heavy chain (right). The CDR3 amino acid sequences of the top 1 BCR clone in each patient were displayed below the UMAP.

Supplementary Fig. S6

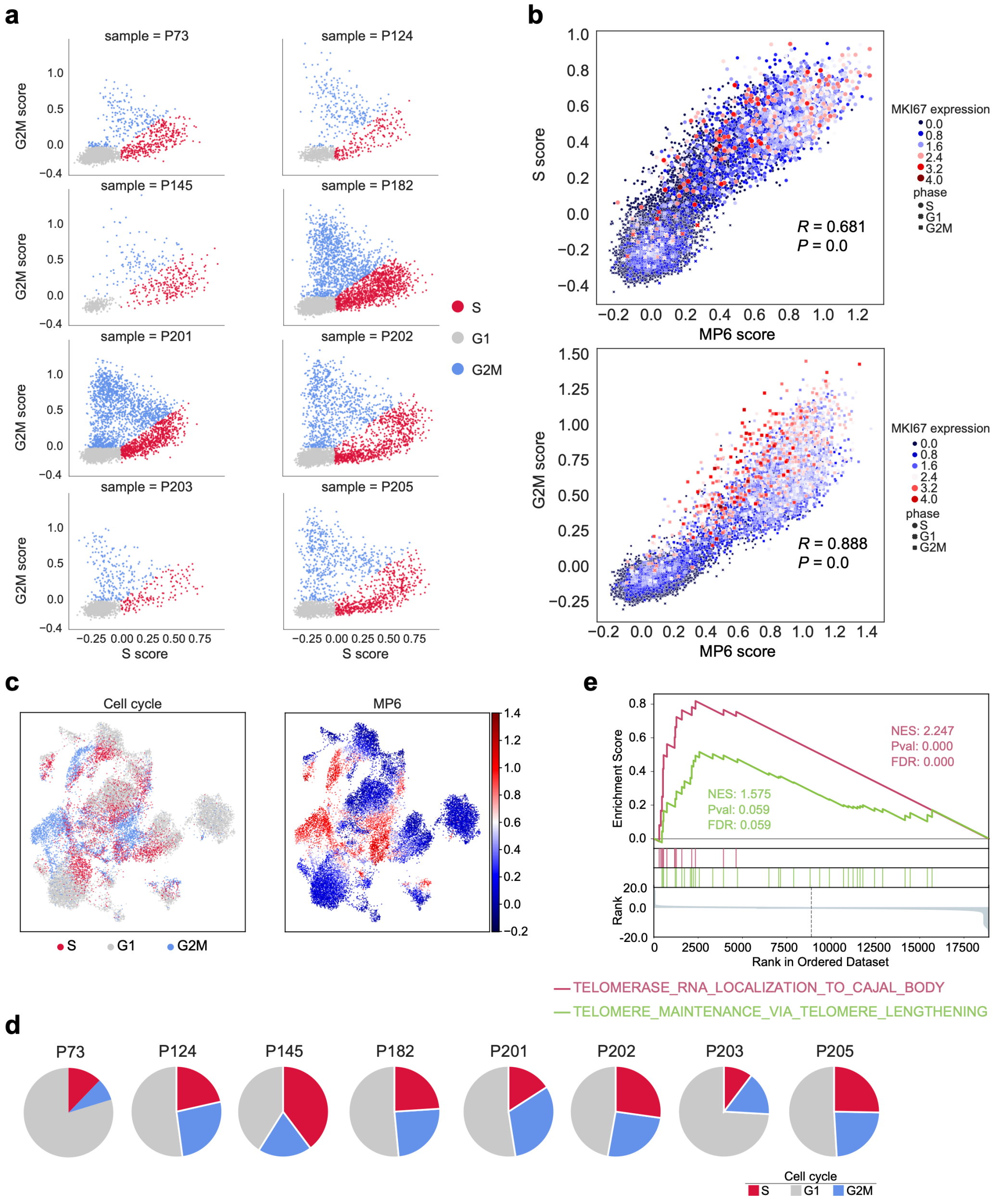

Supplementary Fig. S6 The proliferative characteristics of malignant cells in PCNS DLBCL. a Scatter plot showing the G2M and S score for the malignant B cells of each patient. Cells with positive G2M or S scores were considered to be in cycling state. **b** Scatter plot demonstrating the positive correlation between MP6 signature score and S score (upper) or G2M score (bottom). Both color and size indicated the expression level of MKI67, and dot shape represented cell cycle phases. R, the Pearson correlation coefficient; P, a two-tailed *P*-value for testing non-correlation. **c** UMAP of B cells colored by cell cycle

phases(left) and MP6 signature score (right). **d** Pie plot showing the fraction of cells in different cell cycle phases for each patient. **e** Gene set enrichment analysis (GSEA) for two gene sets associated with telomere maintenance. Genes towards the left were enriched for MP7.

Supplementary Fig. S7

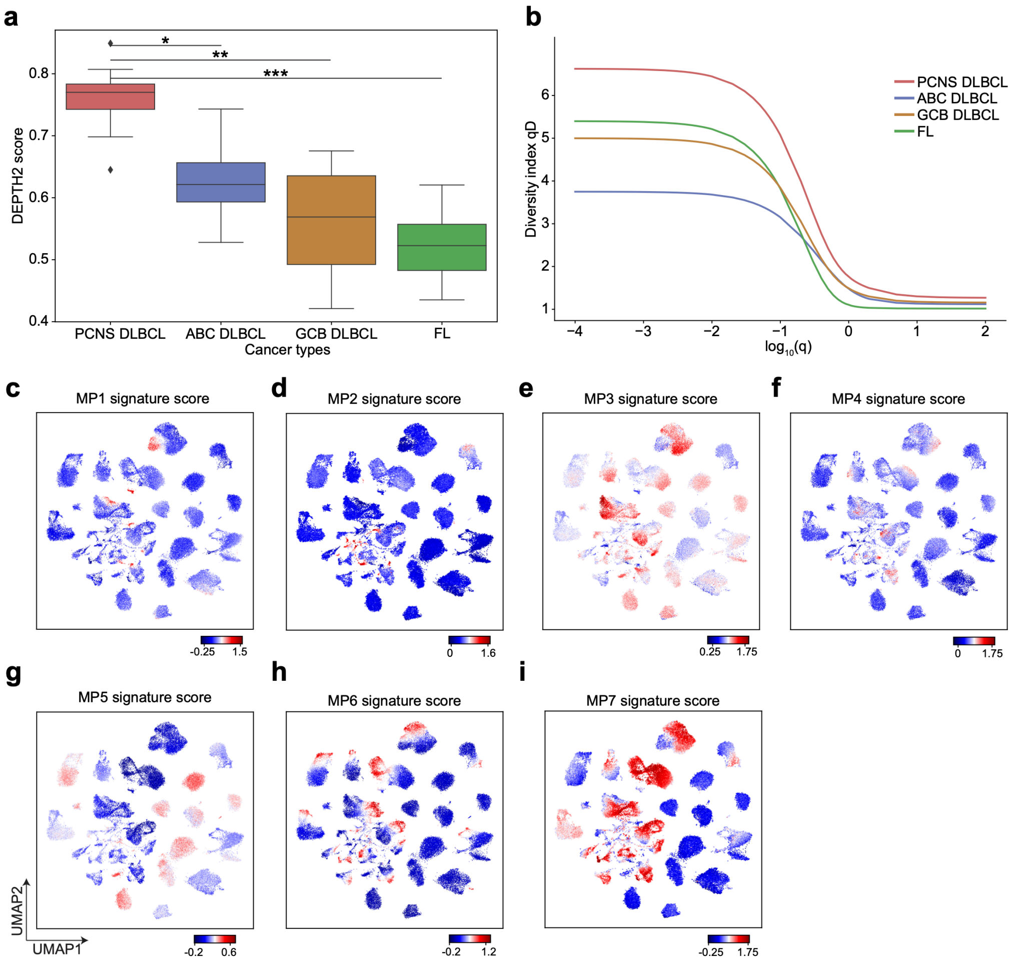

Supplementary Fig. S7 The degree of intratumor heterogeneity and MP signature scores on the integrative malignant B cells. a Boxplot showing the DEPTH2 scores of samples from different cancer types. A two-sided Wilcoxon rank-sum statistic was used to calculate significance (***P < 0.001, **P < 0.01, *P < 0.05). Box boundaries and middle lines correspond to the interquartile range (IQR) and median, respectively. Whiskers extend to the lowest or highest data points that are no more than 1.5 times the IQR from the box boundaries. **b** Line plot of general diversity index across different diversity parameter q values in each cancer type. **c-i** UMAP of malignant B cells colored

by MP signature scores of the seven MPs respectively.

Supplementary Fig. S8

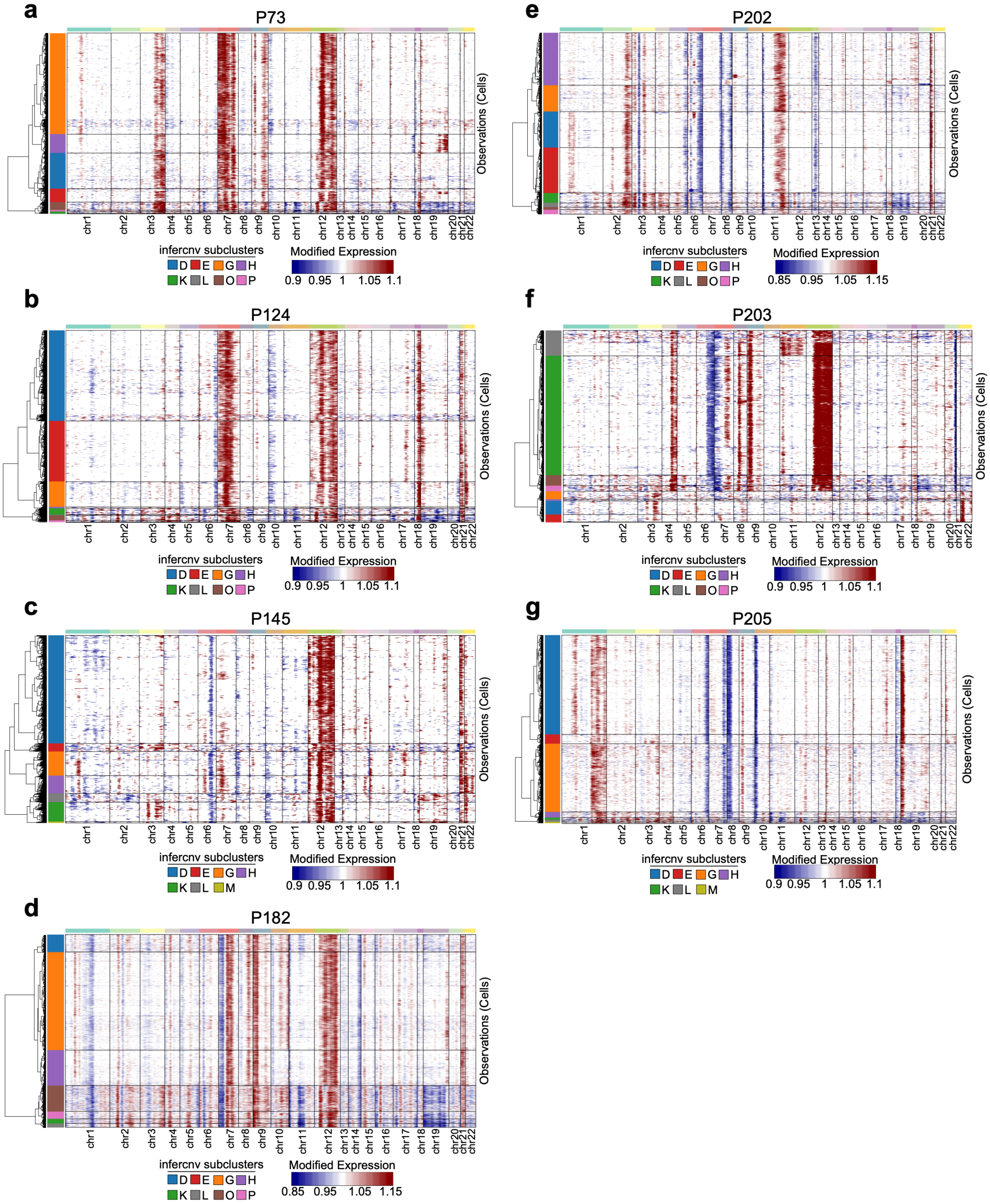

Supplementary Fig. S8 CNV analysis of individual patients. a-g Heatmap of CNV profiles for individual B cells (rows) from each patient, inferred based on the average expression of genes surrounding each chromosomal position (columns). Red: amplifications; blue: deletions.

Supplementary Fig. S9

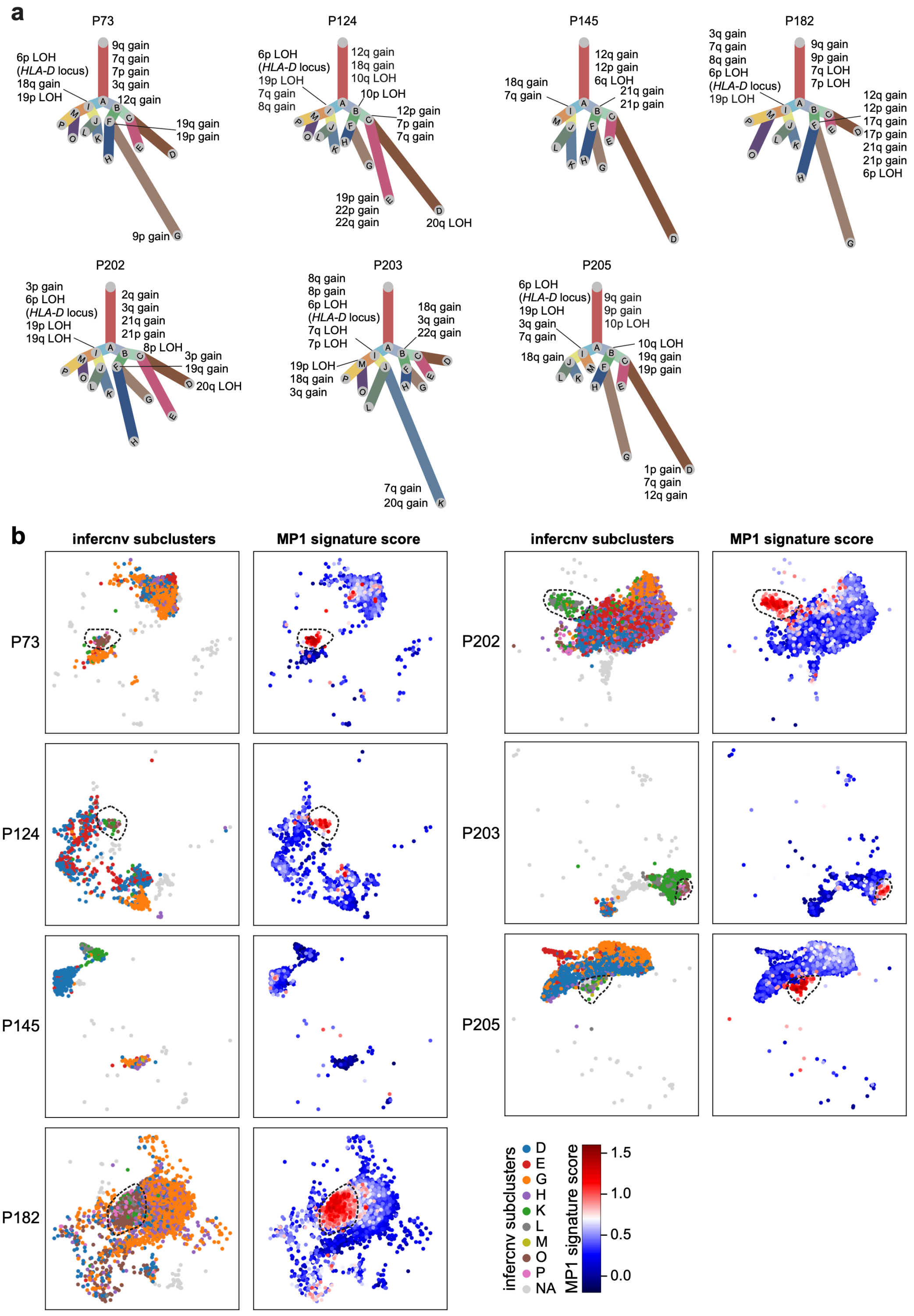

Supplementary Fig. S9 Relationship between MP1 program and inferCNV subclusters across patients. a Clonality trees of the single cells from each patient base on infercnv results. The branches are delineated according to the percentage of cells in the subclone containing the corresponding CNVs. **b**

UMAP of malignant cells in each patient colored by inferCNV subclusters (left) and MP1 signature score (right).

Supplementary Fig. S10

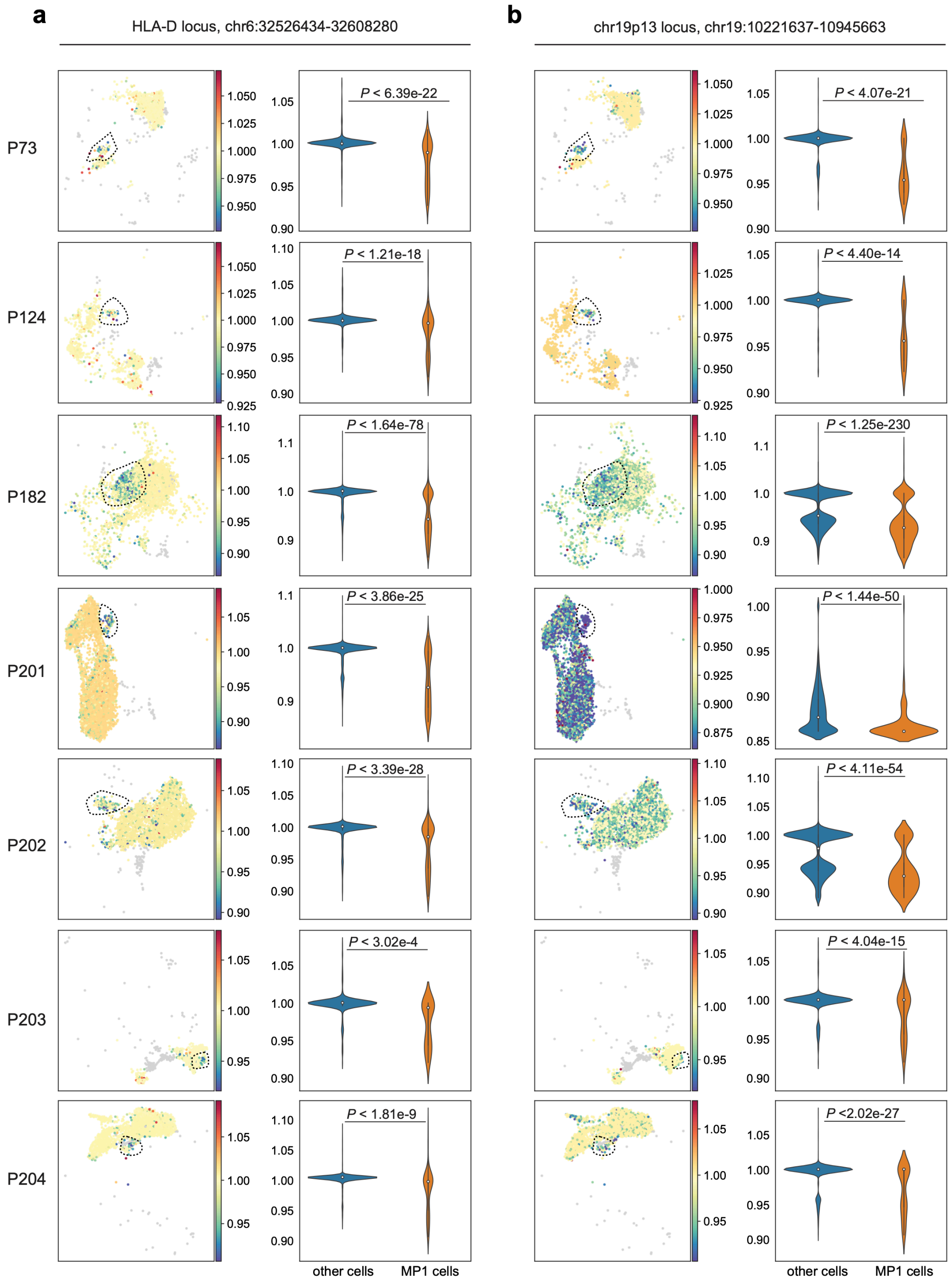

Supplementary Fig. S10 Two recurrent CNV loci in PCNS DLBCL were specifically enriched in MP1 cells. **a** UMAPs of malignant cells in individual samples colored by inferCNV scores of HLA-D locus (left); violin plots of inferCNV scores of HLA-D locus in MP1 cells versus other cells in individual

samples (right). **b** UMAPs of malignant cells in individual samples colored by inferCNV scores of 19p13 locus (left); violin plots of inferCNV scores of 19p13 locus in MP1 cells versus other cells in individual samples (right). In **a** and **b**, a two-sided Wilcoxon rank-sum statistic was used to calculate significance. Notably, cells where MP1 and node I overlap were almost absent in P145. Thus, P145 was not shown.

Supplementary Fig. S11

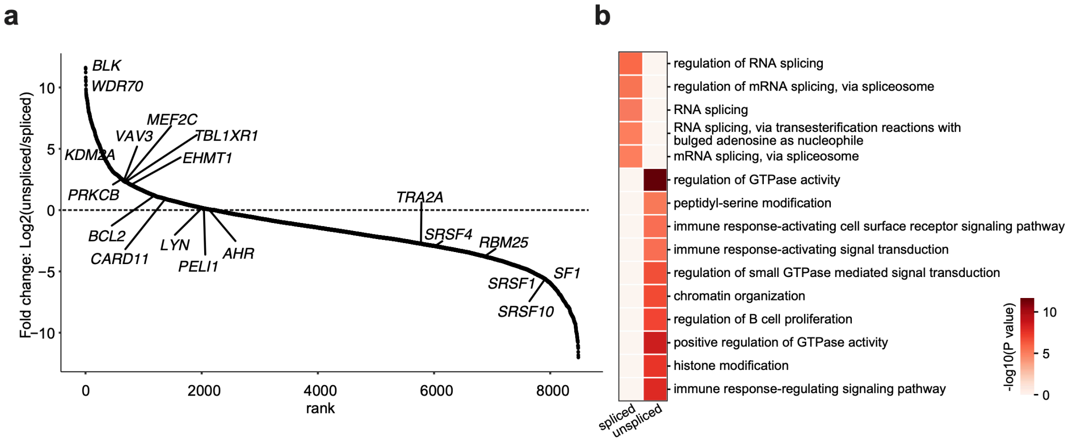

Supplementary Fig. S11 Characterization of spliced and un-spliced genes in node I. a Scatter plot showing the fold changes of the average unspliced counts versus spliced counts of genes in node I cells. **b** Heatmap of representative GO terms enriched in Node I cells in terms of spliced counts and un-spliced counts.

Supplementary Fig. S12

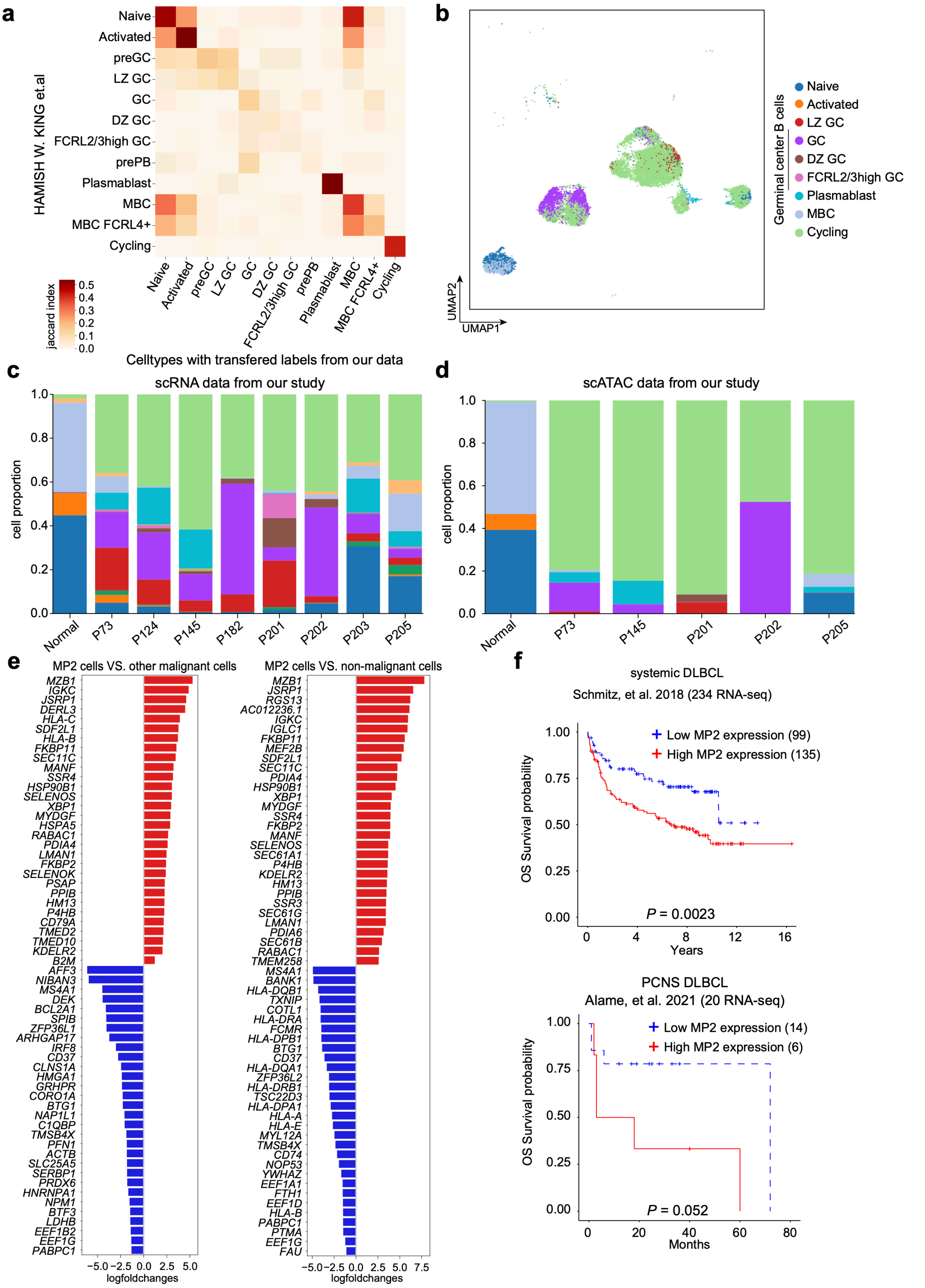

Supplementary Fig. S12 Plasmablast-like malignant cells in PCNS DLBCL. a Jaccard index showing the pairwise correspondence of the top 50 DEGs between B cells clusters in King et al. dataset and those from scRNA-seq in our study. **b** UMAP of malignant cells colored by GC clusters in scATAC data. **c**

Stacked barplot showing the cell proportions of germinal center (GC) clusters in scRNA-seq data. **d** Stacked barplot showing the cell proportions of GC clusters in scATAC-seq data. **e** Barplot of DEGs determined from the comparison between MP2 cells versus other malignant (left) cells and MP2 cells versus non-malignant B cells (right). The top 30 up-regulated and down-regulated DEGs were shown. **f** The overall survival (OS) curve of 234 systemic DLBCL patients (upper) and 20 PCNS DLBCL patients (bottom) based on MP2 signature scores. *P*-value was calculated by log-rank test.

Supplementary Fig. S13

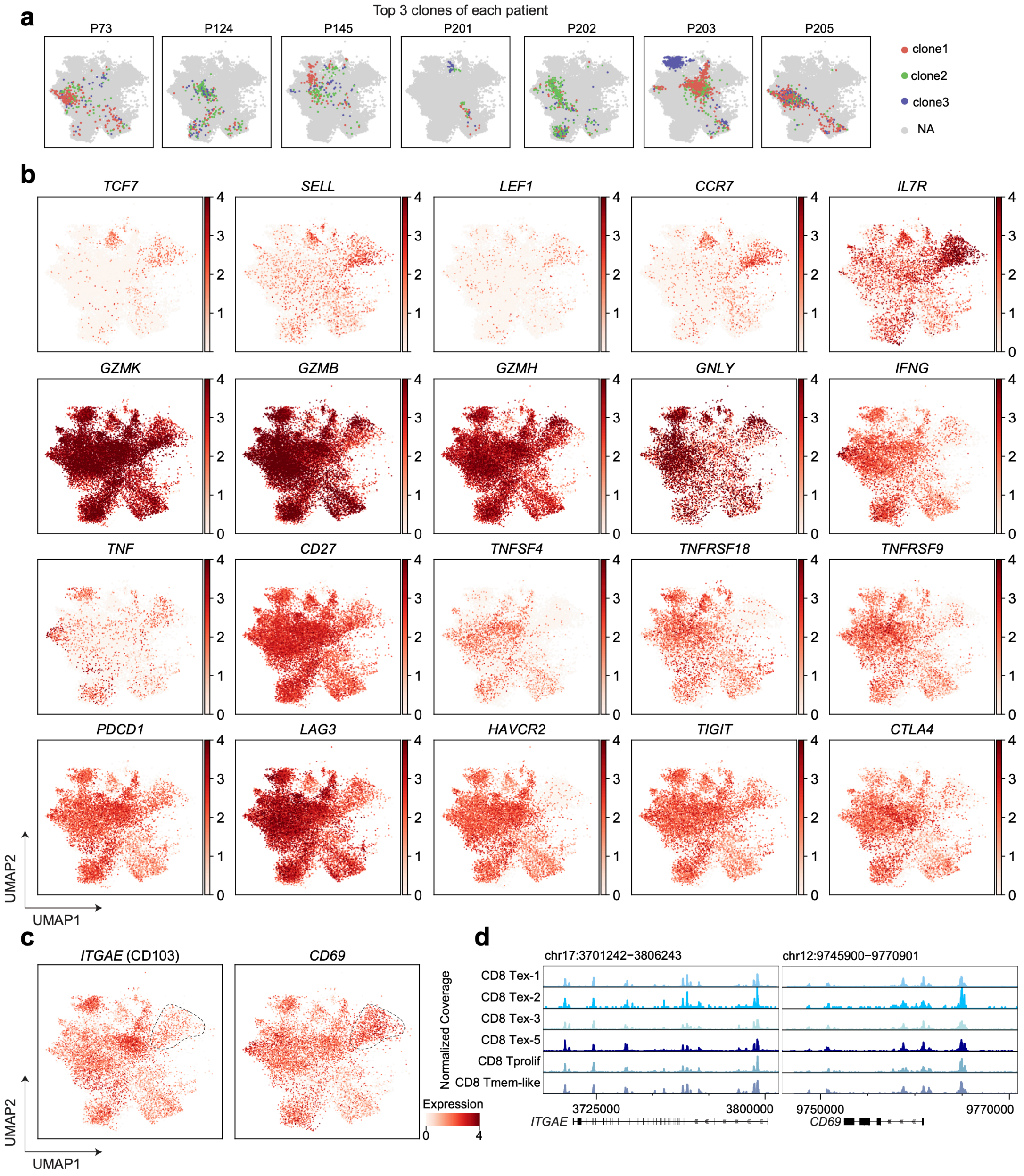

Supplementary Fig. S13 Characterization of bystander CD8 T cells in the TME. a UMAP of CD8 T cells highlighting the top 3 TCR clones in each patient. **b** UMAP of CD8 T cells colored by the normalized expression of selected T-cell functional markers. **c** UMAP of CD8 T cells colored by the expression of ITGAE (CD103) and CD69. **d** Genome tracking plots showing aggregated peaks of ITGAE (CD103) and CD69 for CD8 T clusters in scATAC-seq data.

Supplementary Fig. S14

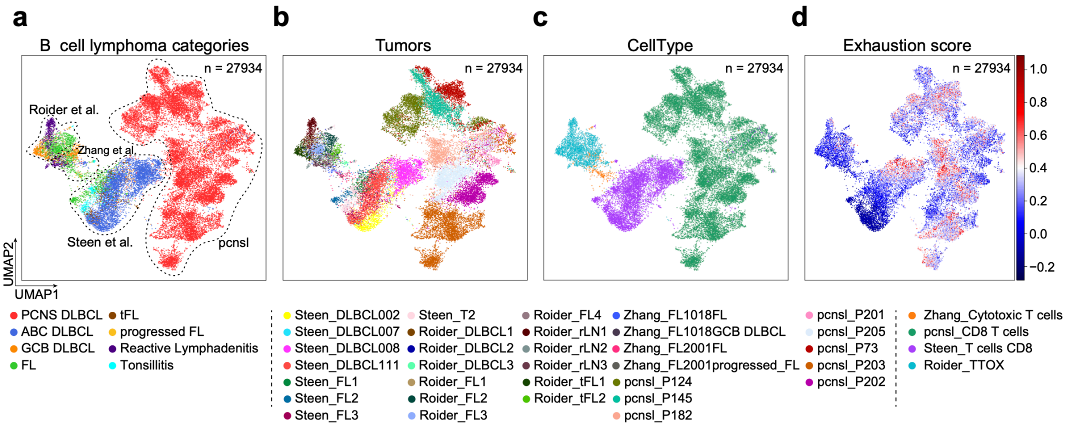

Supplementary Fig. S14 Integrative analysis of CD8 T cells from PCNS DLBCL, systemic DLBCL, follicular lymphoma, Reactive Lymphadenitis, and Tonsillitis cases. a, b, c, d UMAP of CD8 T cells annotated by B-cell lymphoma categories or control cases (**a**), samples (**b**), cluster annotations from the original studies (**c**) and exhaustion score (**d**). In (**a**), dashed circles highlighted the origin of the datasets. FL, follicular lymphoma; progressed FL, progressed follicular lymphoma; tFL, transformed follicular lymphoma. In (**a**), "Steen et al.", "Zhang et al." and "Roider et al." represent three publicly available scRNA-seq datasets in turn: Steen et al.[1], Zhang et al.[2] and Roider et al.[3]; "pcnsl" represents our PCNS DLBCL dataset.

Supplementary Fig. S15

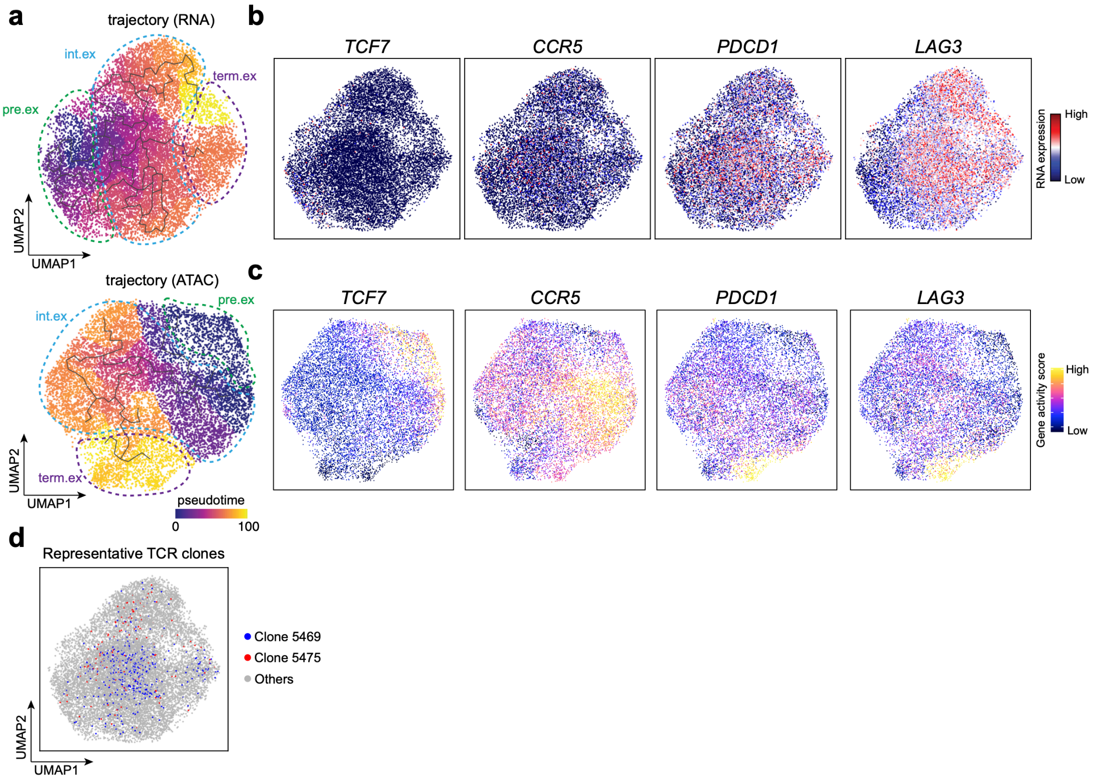

Supplementary Fig. S15 Inference of CD8 T trajectories. a Inferred trajectories of tumor-reactive CD8 T cells of scRNA-seq data (upper) and scATAC-seq data (bottom) colored by pseudotime. **b** UMAP of tumor-reactive CD8 T cells colored by the gene expression (scRNA) of selected markers. **c** UMAP of CD8 T cells colored by the gene activity (scATAC) of selected markers. **d** UMAP of tumor-reactive CD8 T cells colored by representative TCR clones in scRNA data.

Supplementary Fig. S16

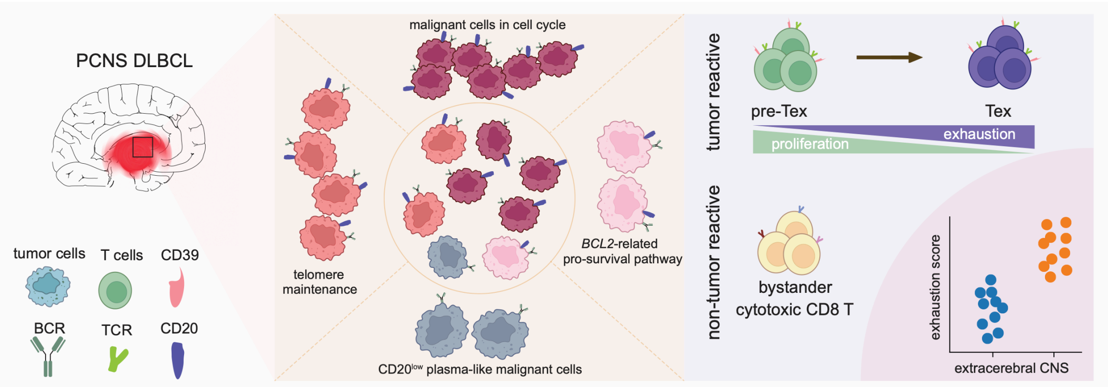

Supplementary Fig. S16 Summary illustration depicting the tumor heterogeneity and immune infiltrates in the TIME of PCNS DLBCL patients.

Malignant B cells varied in expression meta-programs related to cell cycle, pro-survival pathways (including telomere maintenance mechanism, and a PCNS DLBCL-specific program with a pro-survival feature), and plasmablast-like expression program. Moreover, clonally expanded CD8 T cells and a population of bystander CD8 T cells without tumor reactivity coexisted in the TIME. Clonally expanded CD8 T cells were pervasively exhausted resulting from persistent tumor antigen stimulation and showed higher exhaustion scores than systemic DLBCL. In addition, clonally expanded CD8 T cells exhibited a state transition from a pre-exhausted-like to exhausted state across samples.

REFERENCES

1. Steen, C. B. et al. The landscape of tumor cell states and ecosystems in diffuse large B cell lymphoma. *Cancer Cell* **39**, 1422-1437 e10 (2021).
2. Zhang, A. W. et al. Probabilistic cell-type assignment of single-cell RNA-seq for tumor microenvironment profiling. *Nat. Methods* **16**, 1007-1015 (2019).
3. Roider, T. et al. Dissecting intratumour heterogeneity of nodal B-cell lymphomas at the transcriptional, genetic and drug-response levels. *Nat. Cell Biol.* **22**, 896-906 (2020).
